# Learning from multiple annotators for medical image segmentation

**DOI:** 10.1016/j.patcog.2023.109400

**Published:** 2023-06

**Authors:** Le Zhang, Ryutaro Tanno, Moucheng Xu, Yawen Huang, Kevin Bronik, Chen Jin, Joseph Jacob, Yefeng Zheng, Ling Shao, Olga Ciccarelli, Frederik Barkhof, Daniel C. Alexander

**Affiliations:** a Queen Square Institute of Neurology, Faculty of Brain Sciences, University College London, London, WC1B 5EH, United Kingdom; b Centre for Medical Image Computing, Department of Computer Science, University College London, London, WC1E 6BT, United Kingdom; c UCL Respiratory, University College London, London, WC1E 6JF, United Kingdom; d Healthcare Intelligence, Microsoft Research, Cambridge, CB1 2FB, United Kingdom; e Amsterdam UMC, Vrije Universiteit Amsterdam, Department of Radiology and Nuclear Medicine, Amsterdam, Netherlands; f Tencent Jarvis Lab, Shenzhen, China; g Inception Institute of Artificial Intelligence, Abu Dhabi, United Arab Emirates

**Keywords:** Multi-Annotator, Label fusion, Segmentation

## Abstract

Supervised machine learning methods have been widely developed for segmentation tasks in recent years. However, the quality of labels has high impact on the predictive performance of these algorithms. This issue is particularly acute in the medical image domain, where both the cost of annotation and the inter-observer variability are high. Different human experts contribute estimates of the ”actual” segmentation labels in a typical label acquisition process, influenced by their personal biases and competency levels. The performance of automatic segmentation algorithms is limited when these noisy labels are used as the expert consensus label. In this work, we use two coupled CNNs to jointly learn, from purely noisy observations alone, the reliability of individual annotators and the expert consensus label distributions. The separation of the two is achieved by maximally describing the annotator’s “unreliable behavior” (we call it “maximally unreliable”) while achieving high fidelity with the noisy training data. We first create a toy segmentation dataset using MNIST and investigate the properties of the proposed algorithm. We then use three public medical imaging segmentation datasets to demonstrate our method’s efficacy, including both simulated (where necessary) and real-world annotations: 1) ISBI2015 (multiple-sclerosis lesions); 2) BraTS (brain tumors); 3) LIDC-IDRI (lung abnormalities). Finally, we create a real-world multiple sclerosis lesion dataset (QSMSC at UCL: Queen Square Multiple Sclerosis Center at UCL, UK) with manual segmentations from 4 different annotators (3 radiologists with different level skills and 1 expert to generate the expert consensus label). In all datasets, our method consistently outperforms competing methods and relevant baselines, especially when the number of annotations is small and the amount of disagreement is large. The studies also reveal that the system is capable of capturing the complicated spatial characteristics of annotators’ mistakes.

## Abstract (author highlights)

**highlights**
• A novel deep CNN architecture is proposed for jointly learning the expert consensus label and the annotator’s label. The proposed architecture (Fig. 1) consists of two coupled CNNs where one estimates the expert consensus label probabilities and the other models the characteristics of individual annotators (e.g., tendency to over-segmentation, mix-up between different classes, etc) by estimating the pixel-wise confusion matrices (CMs) on a per image basis. Unlike STAPLE [25] and its variants, our method models, and disentangles with deep neural networks, the complex mappings from the input images to the annotator behaviours and to the expert consensus label.
• The parameters of our CNNs are “global variables” that are optimised across different image samples; this enables the model to disentangle robustly the annotators’ mistakes and the expert consensus label based on correlations between similar image samples, even when the number of available annotations is small per image (e.g., a single annotation per image). In contrast, this would not be possible with STAPLE [25] and its variants [5], [8] where the annotators’ parameters are estimated on every target image separately.
• This paper extends the preliminary version of our method presented at the NeurIPS Thirty-fourth Annual Conference on Neural Information Processing Systems [30], by extensively evaluating our model on a new created real-world multiple sclerosis lesion dataset (QSMSC at UCL: Queen Square Multiple Sclerosis Center at UCL, UK). This dataset is generated with manual segmentations from 4 different annotators (3 radiologists with different level skills and 1 expert to generate the expert consensus label). Additionally, we presented a comprehensive discussion about our model’s potential applications (e.g., estimate annotator’s quality and annotation’s quality), the future works we are going to explore, and the potential limitations of our model.

## Graphical abstract

**Figure:**
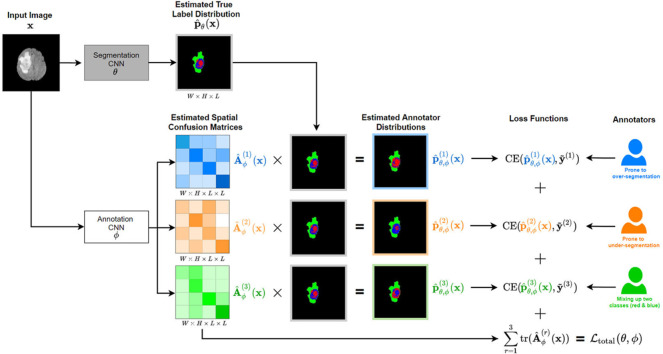

## 1 Introduction

The performance of downstream supervised machine learning models is known to be influenced by substantial inter-reader variability when segmenting anatomical structures in medical images [26]. This issue is especially acute in the medical image domain, where labelled data is commonly scarce due to the high cost of annotations. For instance, because of the heterogeneity in lesion location, size, shape, and anatomical variability across patients [29], accurate identification of multiple sclerosis (MS) lesions in MRIs is difficult even for experienced experts. Another example [21] shows that glioblastoma (a kind of brain tumour) segmentation had an average inter-reader variability of 74–85%. Segmentation annotations of structures in medical image suffer from substantial annotation variations, which is exacerbated by disparities in biases and level of expertise [18]. As a result, despite the current quantity of medical imaging data due to almost two decades of digitisation, the world still lacks access to data with curated labels that can be used by machine learning [14], necessitating the use of intelligent algorithms to learn robustly from such noisy annotations.

Different pre-processing techniques are often used to curate segmentation annotations by fusing labels from different experts in order to minimise inter-reader differences. The most basic and widely used approach is based on a majority vote, with the most representative expert opinion being treated as the expert consensus label. In the aggregation of brain tumour segmentation labels, a smarter variant [21] that accounts for class similarity has proven effective.

However, one major limitation with such approaches is that all experts are presumed to be equally trustworthy[25]. proposed a label fusion approach, which is called STAPLE. This method explicitly models individual expert reliability and uses that knowledge to ”weight” their judgments in the label aggregation step. STAPLE has been the go-to label fusion method in the construction of public medical image segmentation datasets, such as ISLES [27], MSSeg [11], and Gleason’19 [12] datasets, after demonstrating its superiority over traditional majority-vote pre-processing in various applications. Asman further extended this strategy in [4] by accounting for voxel-wise consensus to solve the issue of annotators’ reliability being under-estimated. Another extension [5] was proposed to model the annotator’s reliability across different pixels in images. More recently, STAPLE has been modified in numerous ways to encode the information of the underlying images into the label aggregation process in the context of multi-atlas segmentation problems [2], [16] where image registration is used to warp segments from labelled images (”atlases”) onto a new scan. STEP, which is a way to further incorporate the local morphological similarity between atlases and target images in [8], is a notable example, and several extensions of this approach, such as [1], [6], have subsequently been examined. However, all of the previous label fusion methods have one major limitation: they don’t have a way to integrate information from distinct training images. This severely restricts the scope of applications to situations in which each image has a reasonable number of annotations from multiple experts, which can be prohibitively expensive in practise. Moreover, to model the relationship between observed noisy annotations, expert consensus label and reliability of experts, relatively simplistic functions are utilized, which may fail to capture complex characteristics of human annotators.

**Fig. 1:**
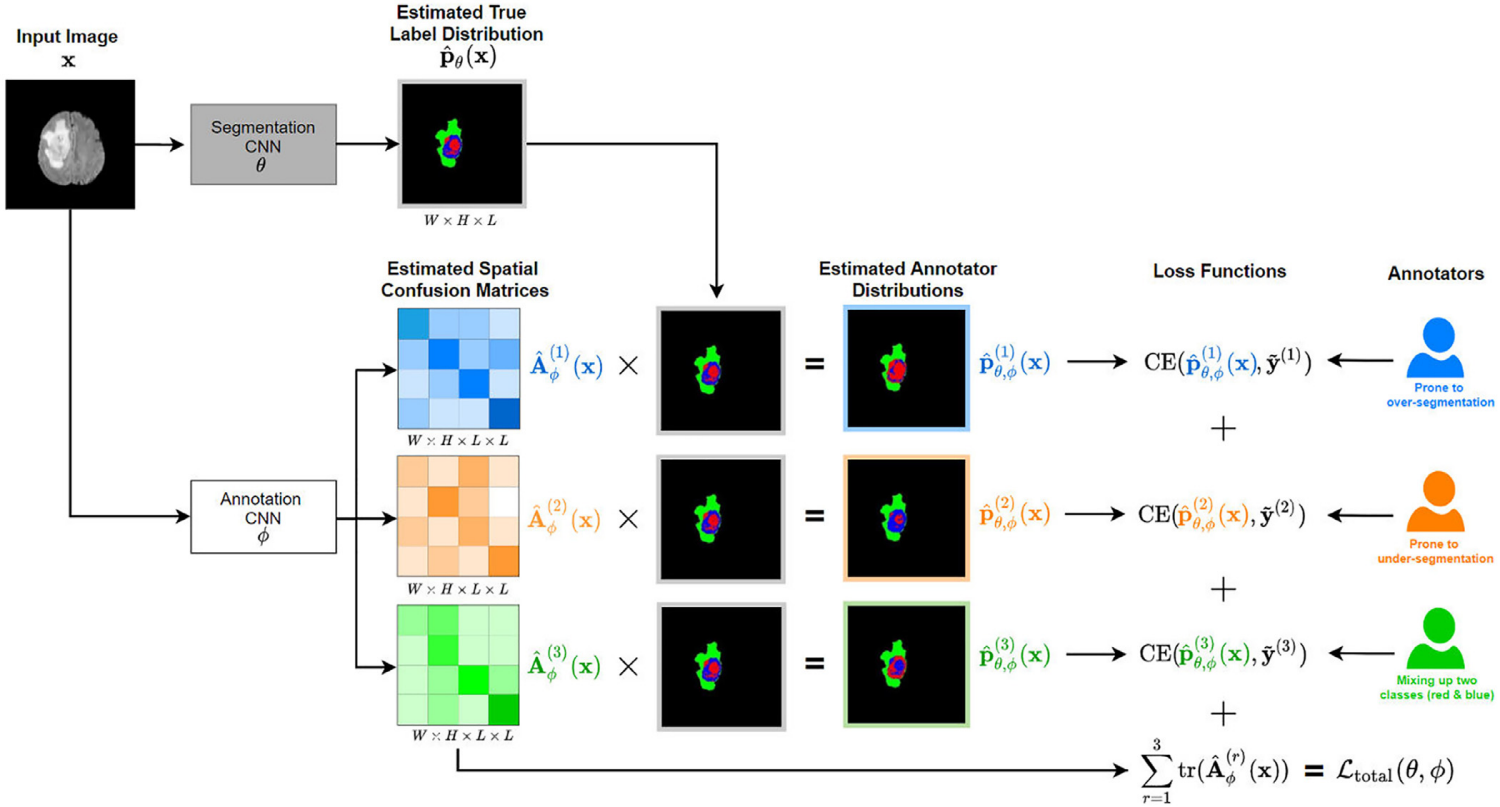
The schematic pipeline of 3 annotators in different characteristics: over-segmentation, under-segmentation and mixing up two classes. The model consists of two parts: (1) *segmentation network* parametrised by θ that generates an estimate of the unobserved expert consensus label probabilities, $p_{\theta }\left(x\right)$; (2) *annotator network*, parametrised by ϕ, that estimates the pixelwise confusion matrices ${\left\{A_{\phi }^{\left(r\right)}\left(x\right)\right\}}_{r=1}^{3}$ of the annotators for the given input image x. During training, the estimated annotators distributions ${\hat{p}}_{\theta ,\phi }^{\left(r\right)}\left(x\right):=A_{\phi }^{\left(r\right)}\left(x\right)\cdot p_{\theta }\left(x\right)$ are computed, and the parameters {θ,ϕ} are learned by minimizing the sum of their cross-entropy losses with respect to the acquired noisy segmentation labels ${\tilde{y}}^{\left(r\right)}$, and the trace of the estimated CMs. At test time, the output of the segmentation network, ${\hat{p}}_{\theta }\left(x\right)$ is used to yield the prediction.

In this paper, we introduce and fully evaluate an unique end-to-end segmentation approach that predicts the reliability of multiple human annotators and the expert consensus label based on noisy labels alone. We use the Morpho-MNIST framework [9] to perform morphometric operations on the MNIST dataset to simulate a variety of annotator types for evaluation. We also demonstrate the potential in several public medical imaging datasets, namely (i) MS lesion segmentation dataset (ISBI2015) from the ISBI 2015 challenge [7], (ii) Brain tumour segmentation dataset (BraTS) [21] and (iii) Lung nodule segmentation dataset (LIDC-IDRI) [3]. Furthermore, we create a practical MS lesion segmentation dataset with 4 different annotators (3 radiologists with different level skills and 1 expert to generate the expert consensus label) to evaluate our model’s performance in real-world data. Experiments on all datasets demonstrate that our method consistently leads to better segmentation performance compared to widely adopted label-fusion methods and other relevant baselines, especially when the number of available labels for each image is low and the degree of annotator disagreement is high. The main contributions of our approach are:

(1) A novel deep CNN architecture is proposed for jointly learning the expert consensus label and the annotator’s label. The proposed architecture (Fig. 1) consists of two coupled CNNs where one estimates the expert consensus label probabilities and the other models the characteristics of individual annotators (e.g., tendency to over-segmentation, mix-up between different classes, etc) by estimating the pixel-wise confusion matrices (CMs) on a per image basis. Unlike STAPLE [25] and its variants, our method models, and disentangles with deep neural networks, the complex mappings from the input images to the annotator behaviours and to the expert consensus label.

(2) The parameters of our CNNs are “global variables” that are optimised across different image samples; this enables the model to disentangle robustly the annotators’ mistakes and the expert consensus label based on correlations between similar image samples, even when the number of available annotations is small per image (e.g., a single annotation per image). In contrast, this would not be possible with STAPLE [25] and its variants [5], [8] where the annotators’ parameters are estimated on every target image separately.

(3) This paper extends the preliminary version of our method presented at the NeurIPS Thirty-fourth Annual Conference on Neural Information Processing Systems [30], by extensively evaluating our model on a new created real-world multiple sclerosis lesion dataset (QSMSC at UCL: Queen Square Multiple Sclerosis Center at UCL, UK). This dataset is generated with manual segmentations from 4 different annotators (3 radiologists with different level skills and 1 expert to generate the expert consensus label). Additionally, we presented a comprehensive discussion about our model’s potential applications (e.g., estimate annotator’s quality and annotation’s quality), the future works we are going to explore, and the potential limitations of our model.

## 2 Methodology

### 2.1 Problem set-up

In this work, we look at the problem of developing a supervised segmentation model using noisy labels provided by multiple human annotators. In particular, we explore a situation in which a set of images ${\left\{x_{n}\in R^{W\times H\times C}\right\}}_{n=1}^{N}$ (with W,H,C denoting the width, height and channels of the image) are assigned with noisy segmentation labels ${\left\{{\tilde{y}}_{n}^{\left(r\right)}\in Y^{W\times H}\right\}}_{n=1,\ldots ,N}^{r\in S(x_{i})}$ from multiple annotators where ${\tilde{y}}_{n}^{\left(r\right)}$ denotes the label from annotator r∈{1,…,R} and $S(x_{n})$ denotes the set of all annotators who labelled image $x_{n}$ and Y=[1,2,…,L] denotes the set of classes.

We suppose that each image x has been annotated by at least one person i.e., |S(x)|≥1, and no expert consensus label ${\left\{y_{n}\in Y^{W\times H}\right\}}_{n=1,\ldots ,N}$ are available. Here the problem of interest comes into *learning the unobserved expert consensus label distribution* p(y∣x) *from such noisy labelled dataset* $D={\left\{x_{n},{\tilde{y}}_{n}^{\left(r\right)}\right\}}_{n=1,\ldots ,N}^{r\in S(x_{n})}$ i.e., the combination of images, noisy labels and labels of experts’ identities (which label was obtained from whom).

We also emphasise that *during inference time, the goal is to segment a particular unlabeled test image,* not to fuse multiple accessible labels, as is typically done in multi-atlas segmentation techniques [16].

### 2.2 Probabilistic model and proposed architecture

In this section, we present the probabilistic model of the observed noisy labels from various annotators. Given the input image, we make two key assumptions: (1) annotators are statistically independent, and (2) annotations over different pixels are independent. With these assumptions, the probability of observing noisy labels ${\left\{{\tilde{y}}^{\left(r\right)}\right\}}_{r\in S(x)}$ on x factorises as:

(1) $$\begin{array}{cc} p({\left\{{\tilde{y}}^{\left(r\right)}\right\}}_{r\in S(x)}|x) & =\prod_{r\in S(x)}p\left({\tilde{y}}^{\left(r\right)}|x\right)\\ & =\prod_{r\in S(x)}\prod_{\begin{array}{c} w\in \{1,\ldots ,W\}\\ h\in \{1,\ldots ,H\} \end{array}}p\left({\tilde{y}}_{wh}^{\left(r\right)}|x\right) \end{array}$$

where ${\tilde{y}}_{wh}^{\left(r\right)}\in \left[1,\ldots ,L\right]$ denotes the ${\left(w,h\right)}^{\text{th}}$ elements of ${\tilde{y}}^{\left(r\right)}\in Y^{W\times H}$. The probability of observing each noisy label on each pixel (w,h) is now rewritten as:

(2) $$p\left({\tilde{y}}_{wh}^{\left(r\right)}|x\right)=\sum_{y_{wh}=1}^{L}p\left({\tilde{y}}_{wh}^{\left(r\right)}|y_{wh},x\right)\cdot p\left(y_{wh}|x\right)$$

where $p(y_{wh}|x)$ denotes the expert consensus label distribution over the ${\left(w,h\right)}^{\text{th}}$ pixel in the image x, and $p({\tilde{y}}_{wh}^{\left(r\right)}|y_{wh},x)$ describes the noisy labelling process by which annotator r corrupts the expert consensus label. In particular, we refer to the L×L matrix whose each ${\left(i,j\right)}^{\text{th}}$ element is defined by the second term $a^{\left(r\right)}{\left(x,w,h\right)}_{ij}:=p\left({\tilde{y}}_{wh}^{\left(r\right)}=i|y_{wh}=j,x\right)$ as the CM of annotator r at pixel (w,h) in image x.

We present a CNN-based architecture for modelling the different constituents of the joint probability distribution in the above $p({\left\{{\tilde{y}}^{\left(r\right)}\right\}}_{r\in S(x)}|x)$ as illustrated in Fig. 1. The model consists of two components: (1) *Segmentation Network*, parametrised by θ, which estimates the expert consensus label probability map, ${\hat{p}}_{\theta }\left(x\right)\in R^{W\times H\times L}$ whose each ${\left(w,h,i\right)}^{\text{th}}$ element approximates $p(y_{wh}=i|x)$;(2) *Annotator Network*, parametrised by ϕ, that generate estimates of the pixel-wise CMs of respective annotators as a function of the input image, ${\left\{{\hat{A}}_{\phi }^{\left(r\right)}\left(x\right)\in {\left[0,1\right]}^{W\times H\times L\times L}\right\}}_{r=1}^{R}$ whose each ${\left(w,h,i,j\right)}^{\text{th}}$ element approximates $p({\tilde{y}}_{wh}^{\left(r\right)}=i|y_{wh}=j,x)$. Each product ${\hat{p}}_{\theta ,\phi }^{\left(r\right)}\left(x\right):={\hat{A}}_{\phi }^{\left(r\right)}\left(x\right)\cdot {\hat{p}}_{\theta }\left(x\right)$ represents the estimated segmentation probability map of the corresponding annotator. Note that here “·” denotes the element-wise matrix multiplications in the spatial dimensions W,H. At inference time, we use the output of the segmentation network ${\hat{p}}_{\theta }\left(x\right)$ to segment test images.

### 2.3 Learning spatial confusion matrices and expert consensus label

Recently, several combined loss functions have been designed and used to solve different problems [13]. In this section, we present the details of how we combined the parameters of the segmentation network, θ, and the parameters of the annotator network, ϕ, to optimise them. In short, we use stochastic gradient descent to minimise the negative log-likelihood of the probabilistic model plus a regularisation component. The following is a more extensive description.

Given training input $X={\left\{x_{n}\right\}}_{n=1}^{N}$ and noisy labels ${\tilde{Y}}^{\left(r\right)}={\left\{{\tilde{y}}_{n}^{\left(r\right)}:r\in S\left(x_{n}\right)\right\}}_{n=1}^{N}$ for r=1,…,R, we optimize the parameters {θ,ϕ} by minimizing the negative log-likelihood (NLL), $-\log p({\tilde{Y}}^{\left(1\right)},\ldots ,{\tilde{Y}}^{\left(R\right)}|X)$. From eqs. 1 and 2, this optimization objective equates to the sum of cross-entropy losses between the observed noisy segmentations and the estimated annotator label distributions:

(3) $$\begin{array}{cc} & -\log p({\tilde{Y}}^{\left(1\right)},\ldots ,{\tilde{Y}}^{\left(R\right)}|X)\\ & \;=\sum_{n=1}^{N}\sum_{r=1}^{R}I\left({\tilde{y}}_{n}^{\left(r\right)}\in S\left(x_{n}\right)\right)\cdot \text{CE}\left({\hat{A}}_{\phi }^{\left(r\right)}\left(x\right)\cdot {\hat{p}}_{\theta }\left(x_{n}\right),\;\;{\tilde{y}}_{n}^{\left(r\right)}\right) \end{array}$$

Keeping the above to a minimum encourages each annotator’s predictions ${\hat{p}}_{\theta ,\phi }^{\left(r\right)}\left(x\right)$ to be as close as feasible to the annotator’s true noisy label distribution $p^{\left(r\right)}\left(x\right)$. This loss function, however, is insufficient to distinguish the annotation noise from the expert consensus label distribution; there are many combinations of pairs ${\hat{A}}_{\phi }^{\left(r\right)}\left(x\right)$ and segmentation model ${\hat{p}}_{\theta }\left(x\right)$ such that ${\hat{p}}_{\theta ,\phi }^{\left(r\right)}\left(x\right)$ perfectly matches the true annotator’s distribution $p^{\left(r\right)}\left(x\right)$ for any input x (e.g., permutations of rows in the CMs). To combat this problem, inspired by [24], which addressed an analogous issue for classification tasks, we add the trace of the estimated CMs to the loss function in Eq. 2.3 as a regularisation term. We thus optimize the combined loss:

(4) $$\begin{array}{cc} L_{\text{total}}\left(\theta ,\phi \right) & :=\sum_{n=1}^{N}\sum_{r=1}^{R}I\left({\tilde{y}}_{n}^{\left(r\right)}\in S\left(x_{i}\right)\right)\\ & \;\cdot \left[\text{CE}\left({\hat{A}}_{\phi }^{\left(r\right)}\left(x\right)\cdot {\hat{p}}_{\theta }\left(x_{n}\right),\;\;{\tilde{y}}_{n}^{\left(r\right)}\right)+\lambda \cdot \text{tr}\left({\hat{A}}_{\phi }^{\left(r\right)}\left(x_{n}\right)\right)\right] \end{array}$$

where S(x)) denotes the set of all labels available for image x, and tr(A) denotes the trace of matrix A. The average probability that a randomly selected annotator would provide an accurate label is represented by the mean trace. Minimizing the trace, on the other hand, encourages the predicted annotators to be as unreliable as possible while minimising the cross entropy ensures fidelity with observed noisy annotators. We minimise this combined loss via stochastic gradient descent to learn both {θ,ϕ}.

### 2.4 Justification for the trace norm

We present a further justification for employing trace regularisation in this section. If the average CM of annotators is *diagonally dominant* and the cross-entropy term in the loss function is zero, [24] demonstrated that minimising the trace of the estimated CMs recovers the true CMs uniquely. However, rather than individual data samples, their results address properties of the average CMs of both the annotators and the classifier over the entire population. In the sample-specific regime, we show a comparable but slightly weaker result, which is more relevant because we estimate CMs of corresponding annotators on every input image.

First, let us set up the notations. For brevity, for a given input image $x\in R^{W\times H\times C}$, we denote the estimated CM of annotator r at ${\left(i,j\right)}^{\text{th}}$ pixel by ${\hat{A}}^{\left(r\right)}:=\left[A^{\left(r\right)}{\left(x\right)}_{ij}\right]\in {\left[0,1\right]}^{L\times L}$. We also define the mean CM $A^{*}:=\sum_{r=1}^{R}\pi_{r}{\hat{A}}^{\left(r\right)}$ and its estimate ${\hat{A}}^{*}:=\sum_{r=1}^{R}\pi_{r}{\hat{A}}^{\left(r\right)}$ where $\pi_{r}\in \left[0,1\right]$ is the probability that the annotator r labels image x. Lastly, as we stated earlier, we assume there is a single expert consensus label per image — thus the true L-dimensional probability vector at pixel (i,j) takes the form of a one-hot vector i.e., $p\left(x\right)=e_{k}$ for, say, class k∈[1,…,L]. Then, the following result motivates the use of the trace regularisation:

Theorem 1 *If the annotator’s segmentation probabilities are perfectly modelled by the model for the given image i.e.,*${\hat{A}}^{\left(r\right)}{\hat{p}}_{\theta }\left(x\right)=A^{\left(r\right)}p\left(x\right)\forall r=1,\ldots ,R$*, and the average true confusion matrix*$A^{*}$at a given pixel and its estimate${\hat{A}}^{*}$satisfy that$a_{kk}^{*}>a_{kj}^{*}$forj≠k*and*${\hat{a}}_{ii}^{*}>{\hat{a}}_{ij}^{*}$for alli,j*such that*j≠i*, then*$A^{\left(1\right)},\ldots ,A^{\left(R\right)}=\text{argmin}{\;}_{{\hat{A}}^{\left(1\right)},\ldots ,{\hat{A}}^{\left(R\right)}}[\text{tr}\left({\hat{A}}^{*}\right)]$*and such solutions are **unique** in the*$k^{\text{th}}$*column where*k*is the correct pixel class.*

The corresponding proof is provided in the supplementary material. The above result shows that if each estimated annotator’s distribution ${\hat{A}}^{\left(r\right)}{\hat{p}}_{\theta }\left(x\right)$ is very close to the true noisy distribution $p^{\left(r\right)}\left(x\right)$ (which is encouraged by minimizing the cross-entropy loss), and for a given pixel, the average CM has the k^th^ diagonal entry larger than any other entries in the same row^2^, then minimizing its trace will drive the estimates of the k^th^ (‘correct class’) columns in the respective annotator’s CMs to match the true values. The single-ground-truth assumption indicates that the remaining values of the CMs are uniformly equal to 1/L, and therefore it suffices to recover the column of the proper class, even though this result is weaker than what was shown in [24] for the population scenario rather than individual samples.

We use identity matrices to encourage $\left\{{\hat{A}}^{\left(1\right)},\ldots ,{\hat{A}}^{\left(R\right)}\right\}$ to be diagonally dominant by training the *annotation network* to maximise the trace for a sufficient number of iterations as a warm-up period. Intuitively, the trace term and cross-entropy combination separates the expert consensus label distribution from the annotation noise by locating the maximum amount of confusion that adequately explains the noisy observations.

### 2.5 Model implementation and optimization

**Low-rank Approximation.** Low-rank approximation is an effective model compression technique to not only reduce parameter storage requirements, but to also reduce computations. For convolutional neural networks (CNNs), however, well-known low-rank approximation methods, such as Tucker or CP decomposition, result in degraded model accuracy because decomposed layers hinder training convergence. We note that each spatial CM, ${\hat{A}}_{\phi }^{\left(r\right)}\left(x\right)$ contains $WHL^{2}$ variables, and calculating the corresponding annotator’s prediction ${\hat{p}}_{\theta ,\phi }^{\left(r\right)}\left(x\right)$ requires WH(2L−1)L floating-point operations, potentially incurring a large time/space cost when the number of classes is large. We also investigate a low-rank approximation (rank=1) approach to alleviate this issue whenever applicable, despite the fact that it is not the focus of our study (as we are concerned with medical imaging applications for which the number of classes is typically limited to less than 10).

Analogous to Chandra and Kokkinos’s work [10] where they employed a similar approximation for estimating the pairwise terms in densely connected CRF, we parametrise the spatial CM, ${\hat{A}}_{\phi }^{\left(r\right)}\left(x\right)=B_{1,\phi }^{\left(r\right)}\left(x\right)\cdot B_{2,\phi }^{T,(r)}\left(x\right)$ as a product of two smaller rectangular matrices $B_{1,\phi }^{\left(r\right)}$ and $B_{2,\phi }^{\left(r\right)}$ of size W×H×L×l where l<<L. In this case, the annotator network outputs $B_{1,\phi }^{\left(r\right)}$ and $B_{2,\phi }^{\left(r\right)}$ for each annotator in lieu of the full CM. Two separate rectangular matrices are used here since the confusion matrices are not necessarily symmetric. Such low-rank approximation reduces the total number of variables to 2WHLl from $WHL^{2}$ and the number of floating-point operations (FLOPs) to WH(4L(l−0.25)−l) from WH(2L−1)L.

**Training without Sample Bias.** Traditionally, machine learning methods can learn model parameters automatically with the training samples and thus it can provide models with good performance which can satisfy the special requirements of various applications. In medical image computing tasks, we usually have the longitudinal study (e.g., our practical MS segmentation data), which is an observational study and the data is gathered from the same sample repeatedly over an extended period of time. Sample bias would occur when our training data only have a limited number of patients from the dataset, which does not reflect the realities of the environment in which a machine learning model will run. For example, certain facial recognition systems are trained primarily on images of white men. These models have considerably lower levels of accuracy with women and people of different ethnicities.

In order to train our model without sample bias and make our model robust to the data generalization, we utilize the product of experts [15] to factor the potential sample biases out of the learned model. Our annotator network is firstly trained with the standard cross-entropy loss from multiple annotators to discover sample biases in the dataset. We then investigate the biases on which the annotator network relies and show that they match the identified sample bias existing in the longitudinal dataset. We also follow the training approaches in [23], which is decomposed into two successive stages: (a) training the annotator network with a standard cross-entropy loss and (b) training the segmentation network via the product of experts to learn from the CMs of the multiple annotators. The core intuition of this training approach is to encourage the robust model to learn to predict the true label distribution that takes into account each annotator’s mistakes (CMs). The final goal is to produce the segmentation network. After training, the annotator network is frozen and used only as part of the product of experts. Since the annotator network is frozen, only the segmentation network receives gradient updates during training.

**Network Details.** With the introduction of CNNs in recent years, image segmentation approaches have improved substantially. CNNs have been applied to both image and model-based segmentation problems with this method outperforming traditional techniques. With regards to the former, the most noticeable breakthrough was the introduction of the U-Net for 2D segmentation by [22]. Subsequently, different variations of the U-Net were proposed, which have extended the method to 3D, dealt with the issue of class imbalance and made full use of the advantages of spatial information. In this work, we implement our model and evaluate on both natural image dataset and medical image datasets in 2D and 3D version based on U-net.

For 2D natural image segmentation tasks, our aim is to study the properties of the proposed model that could estimate the expert consensus label from multiple annotators. Meanwhile, it is also easy to describe and to be understood the theoretical background of our model from the 2D level and suitable to applied on most datasets by exploring this topic on 2D version. For these reasons, we build our model on a 2D U-net [22] with 4 down-sampling stages and channel counts of 32, 64, 128, 256 for each encoder. We also replaced the batch normalisation layers with instance normalisation. Apart from the last layer in the U-net decoder, our segmentation and annotator networks share the same parameters. In essence, the overall architecture is implemented as a U-net with multiple output last layers: one for expert consensus label prediction and the others for noisy segmentation prediction. The output of the last layer of a segmentation network has c channels, where c is the number of classes.

To deal with the more complicated segmentation problems in 3D medical image community, we also implement our model to 3D version. We use the original implementation with some minor modifications. Like our 2D version model, the overall 3D architecture is implemented as a U-net with multiple output last layers: one for expert consensus label prediction and the others for noisy segmentation prediction. In the symmetric encoder path, each layer contains two 3×3×3 convolutions each followed by a rectified linear unit (ReLu), and then a 2×2×2 max pooling with strides of two in each dimension. In the synthesis path, each layer consists of an upconvolution of 2×2×2 by strides of two in each dimension, followed by two 3×3×3 convolutions each followed by a ReLu. Shortcut connections from layers of equal resolution in the analysis path provide the essential high-resolution features to the synthesis path. The last layer that has 1x1x1 kernel and c number of channels as output. In this case, we use ReLu non-linearity and the skip-connections are joined with a concatenation step. The network outputs a c-channel segmentation map with the training labels as well as a softmax.

By default, the output of the last layer for calculating CMs at each spatial position in an annotator network has L×L number of channels; when low-rank approximation is employed, the output of the last layer has 2 L×L number of channels. For fair comparison, we adjusted the number of the channels and the depth of the U-net backbone in Probabilistic U-net [20] to match with our networks.

## 3 Experiments

### 3.1 Dataset description

We evaluate our method on a variety of datasets including both synthetic and real-world scenarios:1) for MNIST segmentation and ISBI2015 MS lesion segmentation challenge dataset [17], we apply morphological operations to generate synthetic noisy labels in binary segmentation tasks; 2) for BraTS 2019 dataset [21], we apply similar simulation to create noisy labels in a multi-class segmentation task; 3) we also consider the LIDC-IDRI dataset which contains multiple annotations per input acquired from different clinical experts as the evaluation in practice. 4) We create a real-world multiple sclerosis lesion dataset with manual segmentations from 4 different annotators to verify our method in practical situation.

### 3.2 Comparison methods and evaluation metrics

Our experiments are based on the assumption that no expert consensus label is available a priori, hence, we compare our method against multiple label fusion methods. In particular, we consider four label fusion baselines: a) mean of all of the noisy labels; b) mode labels by taking the “majority vote”; c) label fusion via the original STAPLE method [25]; d) Spatial STAPLE, a more recent extension of STAPLE that accounts for spatial variations in CMs. For STAPLE and Spatial STAPLE methods, we used the toolkit^3^. To get an upper-bound performance, we also include the *oracle* model that is directly trained on the expert consensus label annotations. To test the value of the proposed image-dependent spatial CMs, we also include “Global CM” model where a single CM is learned per annotator but fixed across pixels and images (analogous to [19], [24], but in segmentation task). Lastly, we also compare against a recent method called Probabilistic U-net [20] as another baseline, which has been shown to capture inter-reader variations accurately.

For evaluation metrics, we use: 1) root-MSE between estimated CMs and real CMs; 2) Dice coefficient (DICE) between estimated segmentation ${\hat{p}}_{\theta }\left(x\right)$ and expert consensus label $y_{GT}$:

(5) $$D_{c}=\frac{2\times \sum_{i}\sum_{j}\left|{\hat{p}}_{\theta }\left(x\right)\cdot y_{GT}\cdot U_{c}\right|}{\sum_{i}\sum_{j}\left|{\hat{p}}_{\theta }\left(x\right)\cdot U_{c}\right|+\sum_{i}\sum_{j}\left|y_{GT}\cdot U_{c}\right|}$$

where $U_{c}$ means the one-hot vector for class c, $U_{c}=\left(U_{1},\ldots ,U_{N}\right),U_{i}=\{\begin{array}{c} 0(i\neq c)\\ 1(i=c) \end{array},c=1,2,\ldots ,N$; 3) The generalized energy distance proposed in [20] to measure the quality of the estimated annotator’s labels. 4) We use the incompetence score, which is defined by calculating the absolute error between the estimated CM and the real CM, to show the learning performance of annotator CNN. 5) We also evaluate our model on both dense labels (multiple labels per image) and single label (randomly selected 1 label per image) in each dataset to show the robustness on sparse labels.

### 3.3 Performance on synthetic datasets

**Fig. 2:**
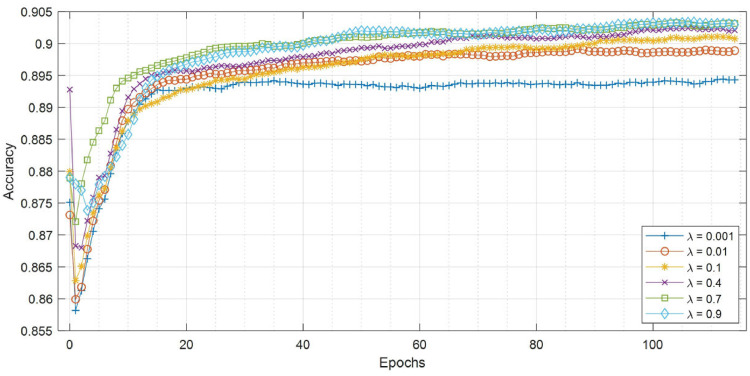
Curves of validation accuracy during training of our model on MNIST for a range of hyperparameters. For our method, the scaling of trace regularizer is varied in [0.001, 0.01, 0.1, 0.4, 0.7, 0.9].).

**Fig. 3:**
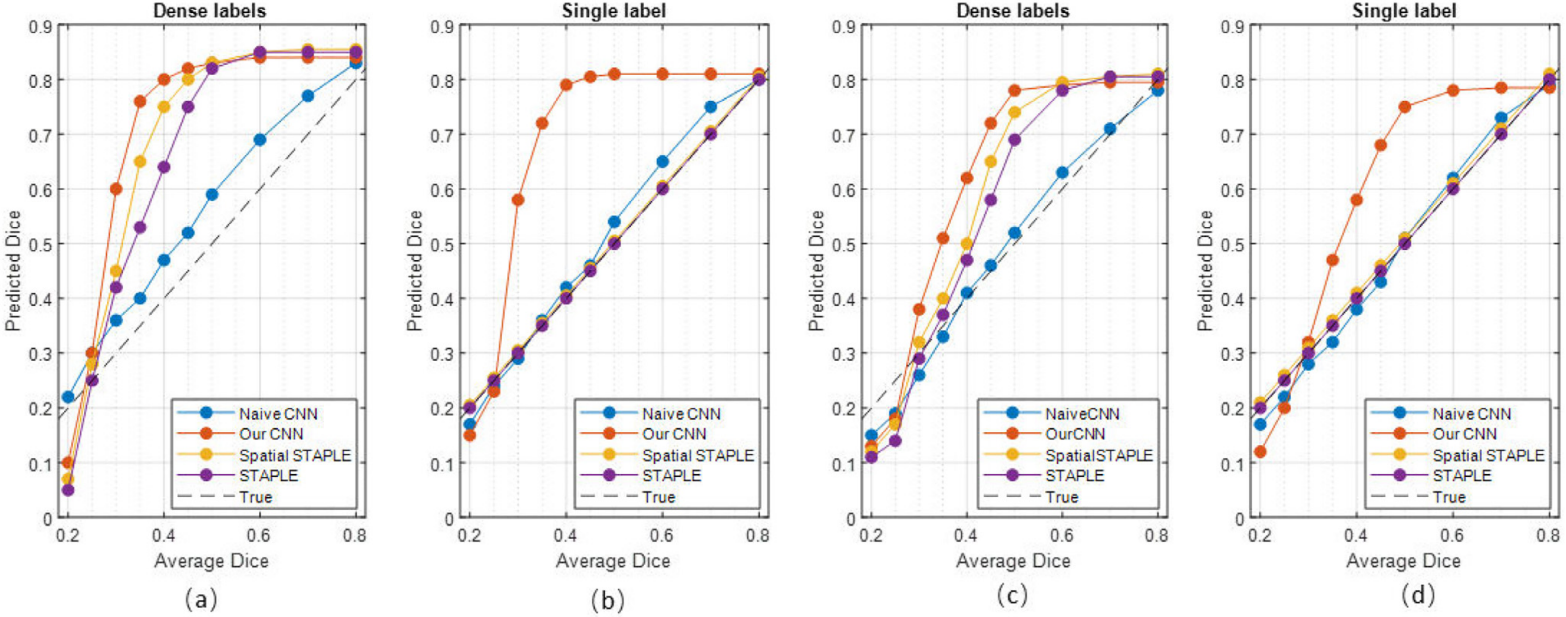
Segmentation accuracy of different models on MNIST (a, b) and MS (c, d) dataset for a range of annotation noise (measured in averaged Dice with respect to expert consensus label.

**MNIST and ISBI2015 Datasets:** On both datasets, our proposed model achieves a higher dice similarity coefficient than STAPLE on the dense label case and, even more prominently, on the single label (i.e., 1 label per image) case (shown in Tables 1 & 2). In addition, our model outperforms STAPLE without or with trace norm, in terms of CM estimation, specifically, we achieve an increase at 6.3%. Additionally, Fig. 2 and Fig. 3 include the performance for different regularisation coefficient and the comparison of the segmentation accuracy on MNIST and ISBI2015 for a range of average dice where labels are generated by a group of 5 simulated annotators.

**Table 1:** Comparison of segmentation accuracy and error of CM estimation for different methods with dense labels (mean ± standard deviation). Numbers in bold indicate the best method that statistically (p<.01) better than other methods by computing the p values of paired t-tests on DICE and CM estimation metrics, respectively.

	MNIST	MNIST	ISBI2015	ISBI2015	
Models	DICE (%)	CM estimation	DICE (%)	CM estimation	
Mean labels	41.94	n/a	42.29	n/a	
Mode labels	58.52	n/a	50.65	n/a	
Naive CNN on mean labels	38.36 ± 0.41	n/a	46.55 ± 0.53	n/a	
Naive CNN on mode labels	62.89 ± 0.63	n/a	47.82 ± 0.76	n/a	
Probabilistic U-net	65.12 ± 0.83	n/a	46.15 ± 0.59	n/a	
Separate CNNs on annotators	70.44 ± 0.65	n/a	46.84 ± 1.24	n/a	
STAPLE	78.03 ± 0.29	0.1241 ± 0.0011	55.05 ± 0.53	0.1502 ± 0.0026	
Spatial STAPLE	78.96 ± 0.22	0.1195 ± 0.0013	58.37 ± 0.47	0.1483 ± 0.0031	
Ours with Global CMs	79.21 ± 0.41	0.1132 ± 0.0028	61.58 ± 0.59	0.1449 ± 0.0051	
Ours without Trace	79.63 ± 0.53	0.1125 ± 0.0037	65.77 ± 0.62	0.1342 ± 0.0053	
Ours	**82.92**±**0.19**	**0.0893**±**0.0009**	**67.55**±**0.31**	**0.0811**±**0.0024**	
Oracle (with known CMs)	83.29 ± 0.11	0.0238 ± 0.0005	78.86 ± 0.14	0.0415 ± 0.0017

**Table 2:** Comparison of segmentation accuracy and error of CM estimation for different methods with one label per image (mean ± standard deviation). Numbers in bold indicate the best method that statistically (p<.01) better than other methods by computing the p values of paired t-tests on DICE and CM estimation metrics, respectively. We note that ‘Naive CNN’ is trained on randomly selected annotations for each image.

	MNIST	MNIST	ISBI2015	ISBI2015
Models	DICE (%)	CM estimation	DICE (%)	CM estimation
Naive CNN	32.79 ± 1.13	n/a	27.41 ± 1.45	n/a
STAPLE	54.07 ± 0.68	0.2617 ± 0.0064	35.74 ± 0.84	0.2833 ± 0.0081
Spatial STAPLE	56.73 ± 0.53	0.2384 ± 0.0061	38.21 ± 0.71	0.2591 ± 0.0074
Ours with Global CMs	59.01 ± 0.65	0.1953 ± 0.0041	40.32 ± 0.68	0.1974 ± 0.0063
Ours without Trace	74.48 ± 0.37	0.1538 ± 0.0029	54.76 ± 0.66	0.1745 ± 0.0044
Ours	**76.48**±**0.25**	**0.1329**±**0.0012**	**56.43**±**0.47**	**0.1542**±**0.0023**

**Fig. 4:**
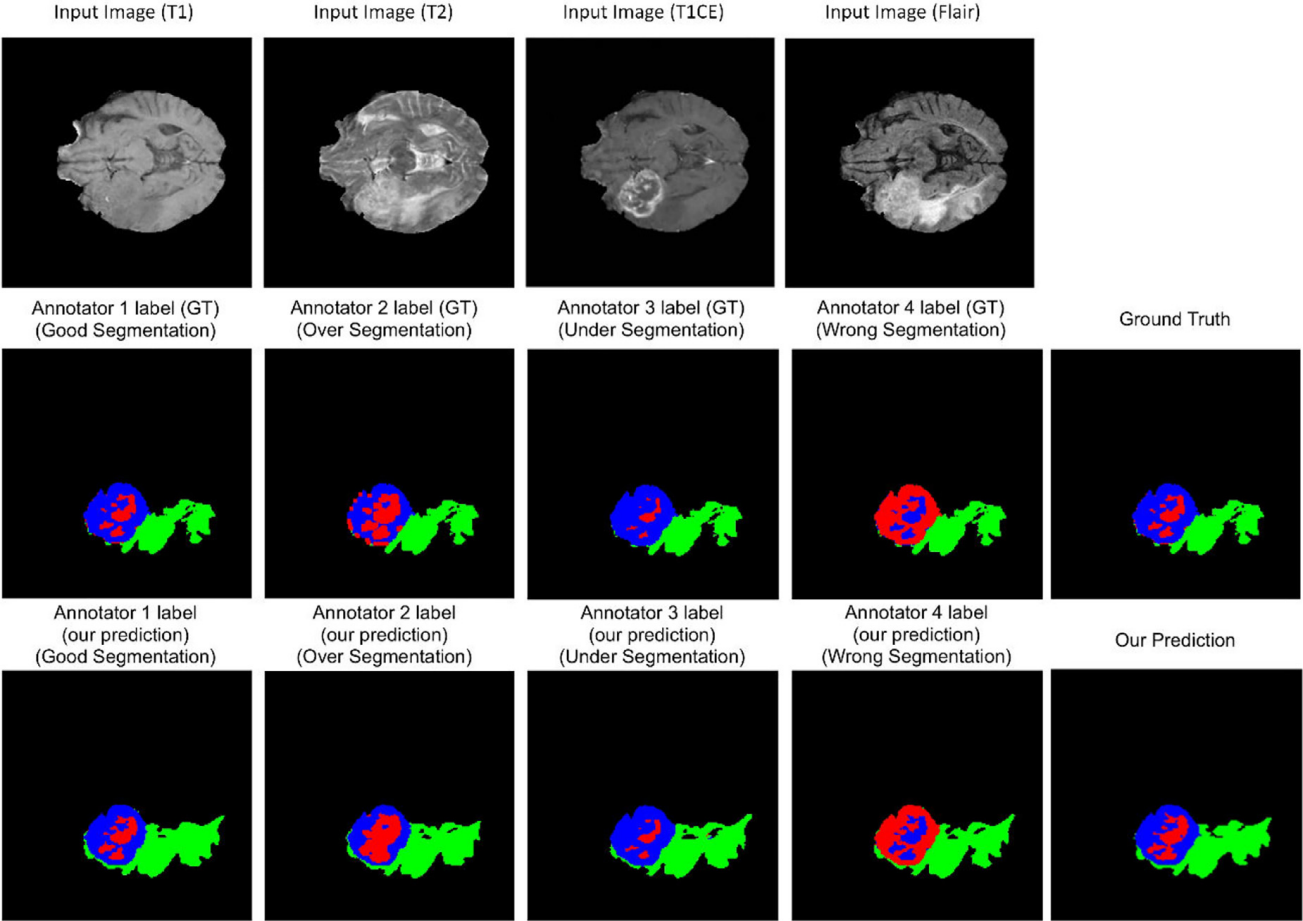
The final segmentation of our model on BraTS and each annotator network predictions visualization. (For interpretation of the references to colour in this figure legend, the reader is referred to the web version of this article.)

**BraTS Dataset:** For multi-class segmentation, our proposed model achieves a higher dice similarity coefficient than STAPLE and Spatial STAPLE on both of the dense labels and single label scenarios (shown in Table 3. In addition, our model outperforms STAPLE in terms of DICE by a large margin at 14.4% on BraTS. In Fig. 4, we visualized the segmentation results and the corresponding annotators’ predictions. Even in multi-class segmentation task, our model can capture each annotator’s characters on annotation.

**Table 3:** Comparison of segmentation accuracy and error of CM estimation for different methods trained with **dense labels** and **single label** (mean ± standard deviation), respectively. For BraTS dataset, we present the results for the target class. Numbers in bold indicate the best method that statistically (p<.01) better than other methods by computing the p values of paired t-tests on DICE and CM estimation metrics, respectively. Note that we count out the Oracle from the model ranking as it forms a theoretical upper-bound on the performance where expert consensus label is known on the training data.

	BraTS	BraTS	BraTS	BraTS
Models	DICE (%)	CM estimation	DICE (%)	CM estimation
	(Dense Labels)	(Dense Labels)	(Single Label)	(Single Label)
Mean labels	34.72	n/a	n/a	n/a
Mode labels	35.74	n/a	n/a	n/a
Naive CNN on mean labels	29.42 ± 0.58	n/a	36.12 ± 0.93	n/a
Naive CNN on mode labels	34.12 ± 0.45	n/a	36.12 ± 0.93	n/a
Probabilistic U-net	40.53 ± 0.75	n/a	n/a	n/a
STAPLE	46.73 ± 0.17	0.2147 ± 0.0103	38.74 ± 0.85	0.2956 ± 0.1047
Spatial STAPLE	47.31 ± 0.21	0.1871 ± 0.0094	41.59 ± 0.74	0.2543 ± 0.0867
Ours with Global CMs	47.33 ± 0.28	0.1673 ± 0.1021	41.76 ± 0.71	0.2419 ± 0.0829
Ours without Trace	49.03 ± 0.34	0.1569 ± 0.0072	43.74 ± 0.49	0.1825 ± 0.0724
Ours	**53.47**±**0.24**	**0.1185**±**0.0056**	**46.21**±**0.28**	**0.1576**±**0.0487**
Oracle (with known CMs)	67.13 ± 0.14	0.0843 ± 0.0029	n/a	n/a

**Fig. 5:**
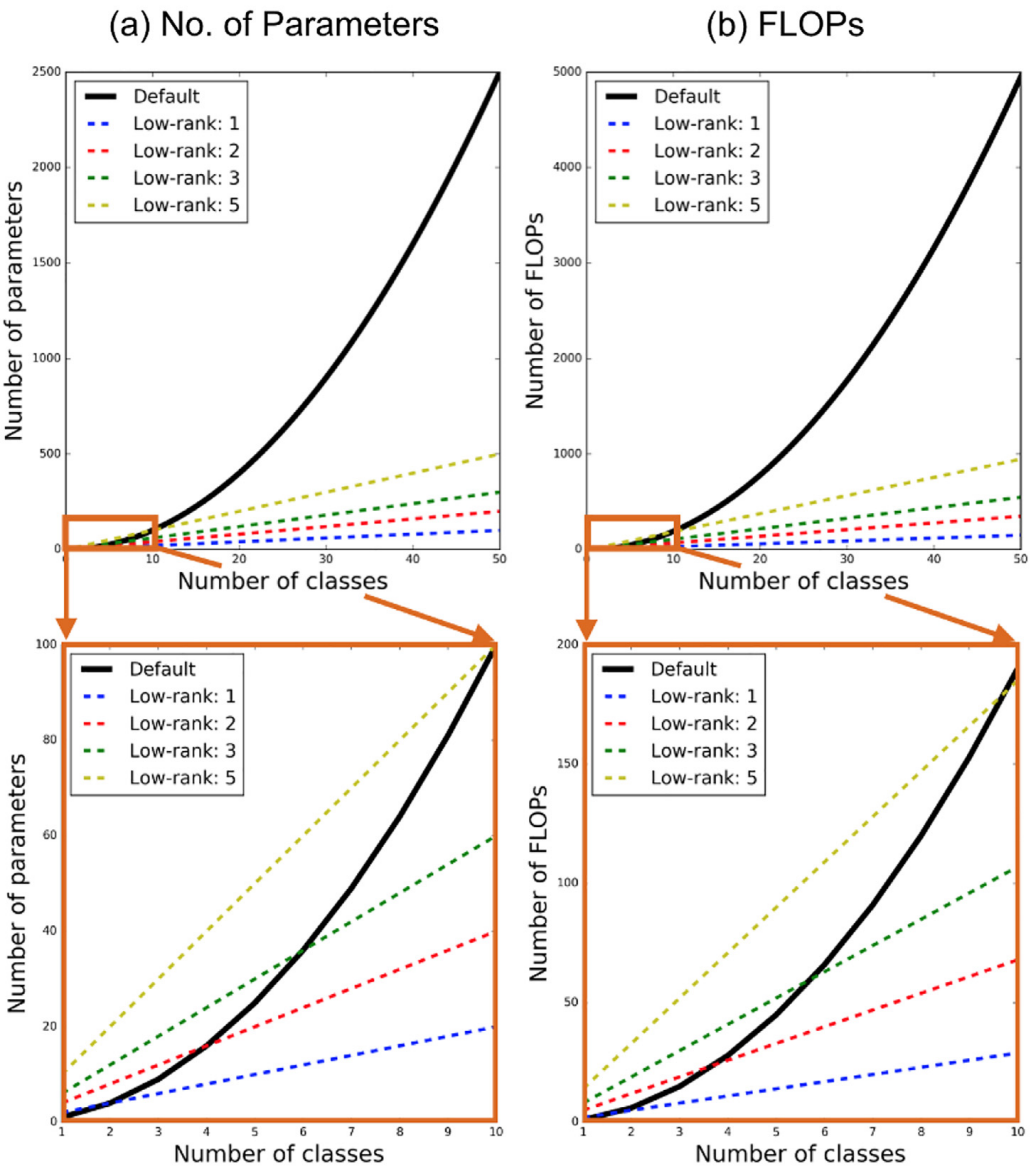
Comparison of time and space complexity between the default implementation and the low-rank counterparts. (a) compares the number of parameters in the confusion matrices while (b) shows the number of FLOPs required to compute the annotator predictions (the product between the confusion matrices and the estimated true segmentation probabilities).

Here we also show our preliminery results on the employed low-rank approximation of confusion matrices for BraTS dataset, precluded in the main text. Table 4 compares the performance of our method with the default implementation and the one with rank-1 approximation. We see that the low-rank approximation can halve the number of parameters in CMs and the number of floating-point-operations (FLOPs) in computing the annotator prediction while resonably retaining the performance on both segmentation and CM estimation. We note, however, the practical gain of this approximation in this task is limited since the number of classes is limited to 4 as indicated by the marginal reduction in the overall GPU usage for one example. We expect the gain to increase when the number of classes is larger as shown in Fig. 5.

**Table 4:** Comparison between the default implementation and low-rank (=1) approximation on BraTS. GPU memory consumption is estimated for the case with batch size = 1. Bot the total number of variables in the confusion matrices, and the number of FLOPs required in computing the annotator predictions.

Rank	Dice	CM estimation	GPU Memory	No. Parameters	FLOPs
Default	53.47 ± 0.24	0.1185 ± 0.0056	2.68GB	589,824	1,032,192
rank 1	50.56 ± 2.00	0.1925 ± 0.0314	2.57GB	294,912	405504

### 3.4 Performance on LIDC-IDRI dataset

**Fig. 6:**
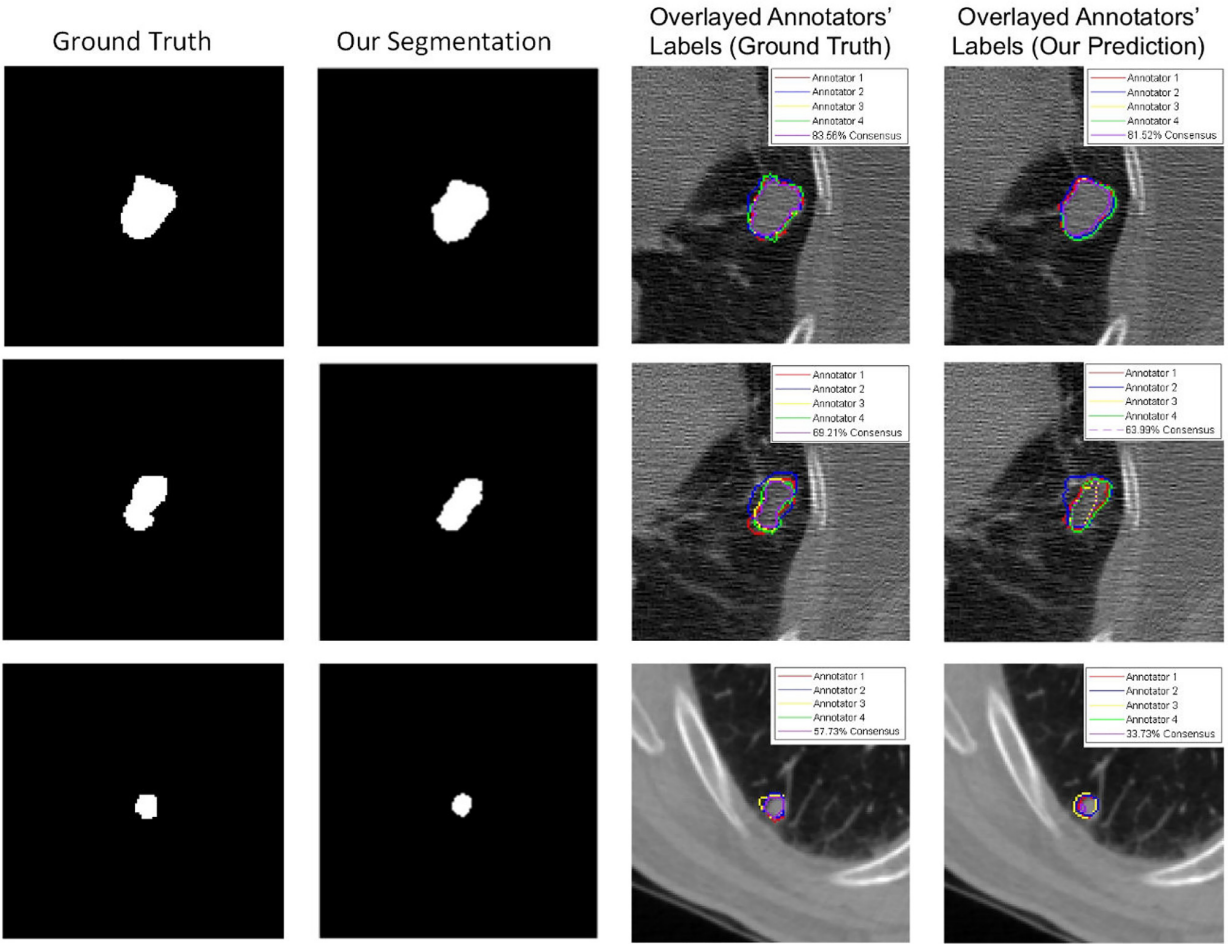
Segmentation results on LIDC-IDRI dataset and the visualization of each annotator contours and the consensus.

In this section, we present our model’s performance on LIDC-IDRI dataset, which has annotation masks generated from 4 radiologists for lesions that they independently detected and considered to be abnormal. Our model (with trace) outperforms STAPLE on single label by a large margin at 18.8%. Since LIDC dataset didn’t provide the annotator identity, we cannot compute the average CM estimation result for each annotator. Thus, we randomly select several samples to visualize the segmentation results and analyse the segmentation performance on different consensus groups. Fig. 6 presents three examples of the segmentation results and the corresponding four annotator contours, as well as the consensus. As shown in the figure, our model successfully predicts both the segmentation of lesions and the variations of each annotator in different cases. We also measure the inter-reader consensus levels by computing the Intersection over Union (IoU) of multiple annotations, and compare the segmentation performance in three subgroups of different consensus levels (low, medium and high).

Additionally, as shown in Table 5, our model consistently outperforms Probabilistic U-Net on generalized energy distance across the four test different datasets, indicating our method can better capture the inter-annotator variations than the baseline Probabilistic U-Net. This result shows that the information about which labels are acquired from whom is useful in modelling the variability in the observed segmentation labels.

**Table 5:** Comparison of Generalised Energy Distance on different datasets (mean ± standard deviation). The distance metric used here is Dice.

Models	MNIST	MS	BraTS	LIDC-IDRI
Prob. U-net [20]	1.46 ± 0.04	1.91 ± 0.03	3.23 ± 0.07	1.97 ± 0.03
Ours	**1.24**±**0.02**	**1.67**±**0.03**	**3.14**±**0.05**	**1.87**±**0.04**

### 3.5 Performance on real-World MS dataset

**Fig. 7:**
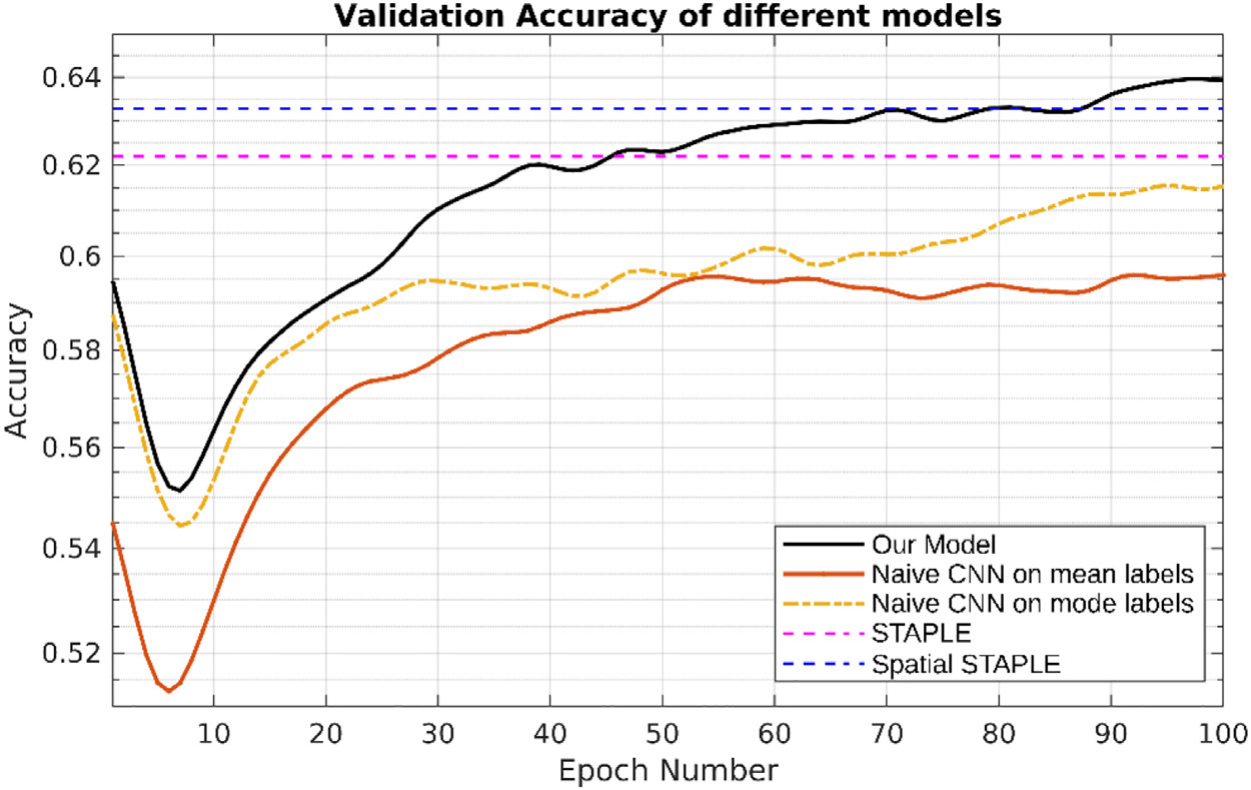
Curves of validation accuracy during training of different models on our practical dataset.

**Fig. 8:**
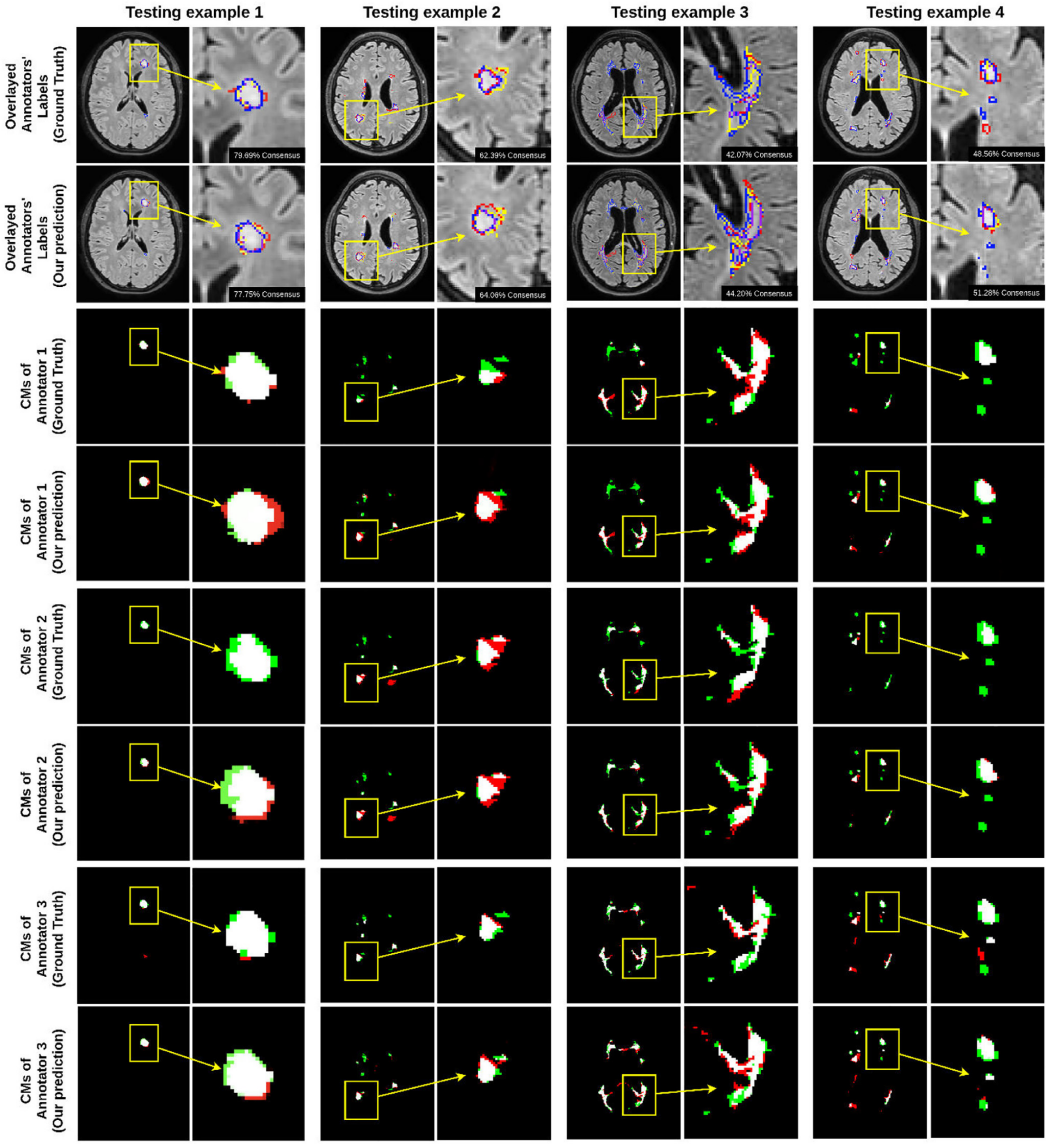
Visualisation of each annotator contours and the consensus (red for Annotator 1, yellow for Annotator 2, blue for Annotator 3 and purple for consensus), and the confusion matrices on our practical dataset (white is the true positive, green is the false negative, red is the false positive and black is the true negative. The background label is learned as true negative and false negative.) (For interpretation of the references to colour in this figure legend, the reader is referred to the web version of this article.)

In Fig. 7 and Table 6, we compared the performance of different methods on our practical dataset. We can see that our model achieved the best result on segmentation accuracy and dice similarity coefficient compared with the state-of-art deep learning based method and the widely used STAPLE, Spatial STAPLE models. We also show the visualization of each annotator contours and the consensus, and the confusion matrices on our practical dataset in Fig. 8. As shown in the figure, our model successfully predicts both the segmentation of lesions and the variations of each annotator in different cases. In the meantime, the confusion matrices in Fig. 8 illustrate our model can capture the patterns of mistakes for each annotator. We also notice that our model is consistently more accurate than the global CM model, indicating the value of image-dependent pixel-wise CMs.

**Table 6:** Comparison of segmentation accuracy and error of CM estimation for different methods trained with **dense labels** (mean ± standard deviation). The best results are shown in bold. Numbers in bold indicate the best method that statistically (p<.01) better than other methods by computing the p values of paired t-tests on DICE and CM estimation metrics, respectively. Note that we count out the Oracle from the model ranking as it forms a theoretical upper-bound on the performance where expert consensus label is known on the training data.

	QSMSC	QSMSC
Models	DICE (%)	CM estimation
Mean labels	40.12	n/a
Mode labels	42.95	n/a
Naive CNN on mean labels	42.31 ± 0.28	n/a
Naive CNN on mode labels	45.84 ± 0.37	n/a
Probabilistic U-net [20]	53.19 ± 0.65	n/a
STAPLE [25]	58.36 ± 0.26	0.3327 ± 0.1026
Spatial STAPLE [5]	61.34 ± 0.29	0.2761 ± 0.1146
Ours with Global CMs	62.08 ± 0.43	0.1869 ± 0.1728
Ours without Trace	63.72 ± 0.72	0.1479 ± 0.0924
Ours	**69.81**±**0.26**	**0.1317**±**0.0769**
Oracle (Ours but with known CMs)	78.49 ± 0.17	0.0715 ± 0.0245

**Fig. 9:**
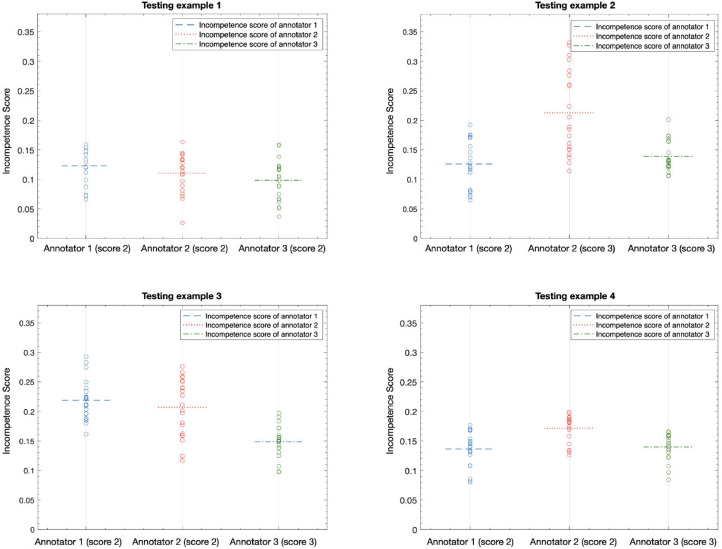
The annotator confidence score of each testing example and corresponding CM incompetence score. We select the middle 20 slices and compute the CM mean error for each example.

**Fig. 10:**
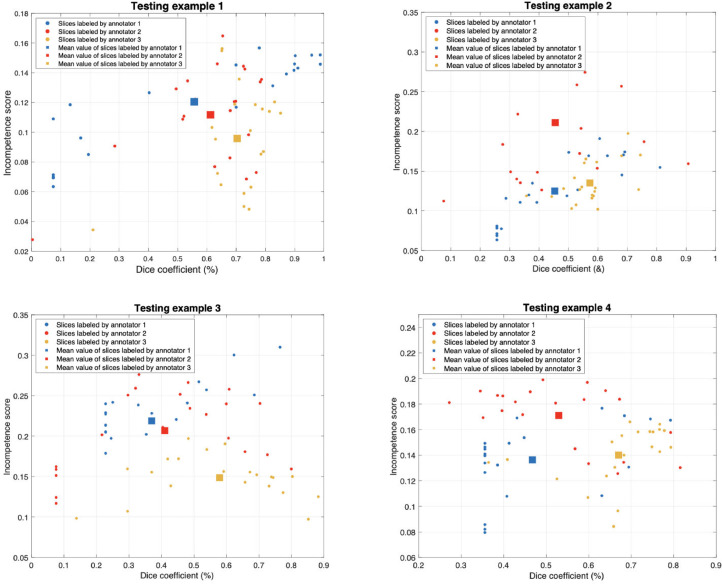
The annotator confidence score of each testing example and corresponding dice coefficient. We select the middle 20 slices and compute the CM mean error for each example.

Furthermore, we show the annotator confidence score of each testing example and corresponding CM errors in Fig. 9. For each annotator, we can tell that if the annotator is not confident on labelling the sample, the lower confidence score will be given and the corresponding CM error will be higher. If different annotators gave the same confidence score for one sample but corresponding incompetence score is different, the annotator who has the lowest incompetence score has the best ability to label the data. For example, annotator 3 labelled the four testing examples with high confidence and the learned CMs show the lowest incompetence score, we can consider annotator 3 has the best ability to label the data. To further verify the annotator who has the best annotation ability, we show the correlation between annotator’s confidence score and corresponding dice coefficient for each testing example in Fig. 10. We can see that the annotator 3 also has the best performance on dice coefficient but with lowest CM incompetence score for each testing example. From both figures, we can tell the annotator 3 has the best ability to give labels for the MS lesions.

## 4 Discussion

In this work, we integrate two coupled CNNs into an end-to-end supervised segmentation framework to jointly estimate the reliability of multiple human annotators and expert consensus label from noisy labels alone, which is applicable to different medical image segmentation tasks. Our method is very lightweight and can be trained in an end-to-end manner. In the following, we present a comprehensive discussion for some questions we are concerning in this work and the future extension of our model, such as the potential application on the education of teaching people how to label the image data and selecting the best annotator from multiple annotators.

### 4.1 Evaluation on 3D multi-class segmentation

For most multi-class image segmentation problems, the number of pixels in each class is different from each other which potentially leads to less accurate predictions for some classes than others. Additionally, some of the image regions are easier to be classified (i.e. higher segmentation accuracy) than others due to more distinct local image characteristics. In our work, to validate the synthetic noisy labels in multi-class segmentation for 3D medical images, e.g., BraTS dataset, we choose a target class and treat the other classes as ”background”. In Table 3 & 4, we only present the dice coefficient and the CM estimation for the target class for Brats image. To validate the segmentation model’s performance, we also show the dice coefficient for all classes and the entire image $D_{total}$ by judging prediction is correct or incorrect with Eq. 6:

(6) $$D_{total}=\frac{2\times \sum_{i}\sum_{j}\left|{\hat{p}}_{\theta }\left(x\right)\cdot y_{GT}\right|}{\sum_{i}\sum_{j}\left|{\hat{p}}_{\theta }\left(x\right)\right|+\sum_{i}\sum_{j}\left|y_{GT}\right|}$$

Quantitative results from the comparison models are presented in Table 7 and 8. Our model show the best performance in all presented methods. The segmentation accuracy was improved roughly 14% and 17% compared to STAPLE and Spatial STAPLE in dense labels and single label, respectively. This means that our model worked well for capturing the annotator’s character even with multi-class lesions in the images.

**Table 7:** Dice coefficients for multi-class segmentation results with different comparison models trained with dense labels per image (mean ± standard deviation). *Average Dice* is the average of each class’s dice coefficient, *Total Dice* is the average of entire image dice coefficient. Numbers in bold indicate the best method that statistically (p<.01) better than other methods by computing the p values of paired t-tests on DICE metric.

	Class 1	Class 2	Class 3	Average Dice	Total Dice
Models	(Necrosis: Target)	(Enhancing)	(Edema)	(All Classes)	(Entire Image)
Mean labels	34.72	32.72	36.97	34.80	69.54
Mode labels	35.74	33.69	38.36	35.93	71.32
Naive CNN on mean label	29.42 ± 0.58	25.26 ± 0.42	32.87 ± 0.29	29.18 ± 0.43	73.85 ± 0.36
Naive CNN on mode label	34.12 ± 0.45	31.95 ± 0.33	39.27 ± 0.41	35.11 ± 0.39	75.67 ± 0.42
Probabilistic U-net	40.53 ± 0.75	38.69 ± 0.28	44.12 ± 0.38	41.11 ± 0.47	80.69 ± 0.52
STAPLE	46.73 ± 0.17	45.38 ± 0.32	48.59 ± 0.24	46.90 ± 0.24	81.28 ± 0.34
Spatial STAPLE	47.31 ± 0.21	48.11 ± 0.34	48.67 ± 0.25	48.03 ± 0.27	84.39 ± 0.47
Ours with Global CMs	47.33 ± 0.28	48.36 ± 0.41	49.71 ± 0.33	48.47 ± 0.34	85.14 ± 0.33
Ours without Trace	49.03 ± 0.34	47.59 ± 0.49	52.34 ± 0.31	49.65 ± 0.38	85.81 ± 0.49
Ours	**53.47**±**0.24**	**51.94**±**0.51**	**55.69**±**0.42**	**53.70**±**0.39**	**87.24**±**0.32**
Oracle (with known CMs)	67.13 ± 0.14	66.02 ± 0.23	68.85 ± 0.17	67.33 ± 0.18	90.18 ± 0.25

**Table 8:** Dice coefficients for multi-class segmentation results with different comparison models trained with only one label available per image (mean ± standard deviation). *Average Dice* is the average of each class’s dice coefficient, *Total Dice* is the average of entire image dice coefficient. Numbers in bold indicate the best method that statistically (p<.01) better than other methods by computing the p values of paired t-tests on DICE metric.

	Class 1	Class 2	Class 3	Average Dice	Total Dice
Models	(Necrosis: Target)	(Enhancing)	(Edema)	(All Classes)	(Entire Image)
Naive CNN	36.12 ± 0.93	35.62 ± 0.74	39.64 ± 0.58	37.13 ± 0.75	76.34 ± 0.52
STAPLE	38.74 ± 0.85	38.02 ± 0.92	40.62 ± 0.67	39.13 ± 0.81	78.39 ± 0.57
Spatial STAPLE	41.59 ± 0.74	40.37 ± 0.68	43.59 ± 0.72	41.85 ± 0.71	80.26 ± 0.64
Ours with Global CMs	41.76 ± 0.71	41.38 ± 0.59	44.62 ± 0.71	42.59 ± 0.67	80.73 ± 0.51
Ours without Trace	43.74 ± 0.49	42.29 ± 0.61	45.87 ± 0.49	43.97 ± 0.53	84.69 ± 0.57
Ours	**46.21**±**0.28**	**45.76**±**0.38**	**48.91**±**0.34**	**46.96**±**0.33**	85.26 ± 0.27

### 4.2 Learn Annotator’s quality

In the experiments on practical MS dataset, we measure each annotator’s incompetence score of the confusion matrices, confidence score for the annotation and the computed dice coefficient. In Fig. 9 and 10, we plot the correlations of the CM incompetence score vs. the annotator confidence score and the dice coefficient, respectively. This is done for the testing cases, which we selected the middle 20 slices for each and annotated by the 3 different annotators. From Fig. 9 we can tell that the more confident the annotator, the smaller CM incompetence score, so we could choose the annotator 3 as the best one in this stage. Furthermore, we calculated the mean dice coefficient of the selected slices for each testing case. From the results shown in Fig. 10, we can tell that annotator 3 still show the best performance with higher dice coefficient and smaller CM incompetence score. Overall, by computing the three criterias, we could rank the practical annotators’ ability on data labelling (Annotator1<Annotator2<Annotator3 in this work).

### 4.3 Learning with metadata

Metadata is an useful and powerful machine learning tool to be collected in any data scientists’ toolbox, regardless of the model we are using. Unfortunately, there is a paucity of quality literature on this topic and metadata is often overlooked when building a accurate machine learning model. In this work, the metadata can include information about each annotator’s experience, fatigue, motivation, concentration. For example, annotator’s experience (e.g., expert, senior, junior) is different for different types of lesions, which require different levels and types of expertise. The different annotation experience also affect annotation quality and availability of a worker base. As for the setup, annotator motivation is also one of the key aspects determining the cost of annotations. In the future work, to improve the segmentation accuracy, we plan to integrate all the available metadata in the proposed method.

## 5 Conclusion

We introduced a novel, robust learning method based on CNNs for simultaneously recovering the label noise of multiple annotators and the expert consensus label distribution for supervised segmentation problems. We demonstrated this method on real-world datasets with synthetic annotations and real-world annotations. Our method is capable of estimating individual annotators and thereby improving robustness against label noise. Experiments have shown our model achieves considerable improvement over the traditional label fusion approaches including averaging, the majority vote and the widely used STAPLE framework and spatially varying versions, in terms of both segmentation accuracy and the quality of CM estimation.

One exciting avenue of this research is the application of the annotation models in downstream tasks. Of particular interest is the design of active data collection schemes where the segmentation model is used to select which samples to annotate (“active learning”), and the annotator models are used to decide which experts to label them (“active labelling”)—e.g., extending [28] from simple classification task to segmentation remains as the future work. Another exciting application is education of inexperienced annotators; the estimated spatial characteristics of segmentation mistakes provide further insights into their annotation behaviours, and as a result, help them improve the quality of their annotations in the next data acquisition. At the same time, although we have achieved reliable performance on all experiments, it is worth to explore the question that how many training samples at least to achieve the reliable performance for each annotator. This is also a future work we will consider.

## Declaration of Competing Interest

The authors certify that they have NO affiliations with or involvement in any organisation or entity with any financial interest (such as honoraria; educational grants; participation in speakers’ bureaus; membership, employment, consultancies, stock ownership, or other equity interest; and expert testimony or patent-licensing arrangements), or non-financial interest (such as personal or professional relationships, affiliations, knowledge or beliefs) in the subject matter or materials discussed in this manuscript.

## Data Availability

Data will be made available on request.

## Appendix A Supplementary materials

Supplementary Data S1 Supplementary Raw Research Data. This is open data under the CC BY license http://creativecommons.org/licenses/by/4.0/ Supplementary Data S1

## Footnotes

2 For the standard “majority vote” label to capture the correct expert consensus label, one requires the k^th^ diagonal element in the average CM to be larger than the sum of the remaining elements in the same row, which is a more strict condition.

3 https://www.nitrc.org/projects/masi-fusion/
