# Supplementary material for: Learning from multiple annotators for medical image segmentation
Source: Pattern Recognit. 2023 Jun;138:None. doi: 10.1016/j.patcog.2023.109400 (PMC10533416; doi:10.1016/j.patcog.2023.109400)
Supplement: Supplementary Data S1 — Supplementary Raw Research Data. This is open data under the CC BY license http://creativecommons.org/licenses/by/4.0/ [file mmc1.pdf]

Supplementary Material for

Learning to Segment from Multiple Annotators

6. Datasets Description

MNIST Segmentation Dataset: We define a MNIST segmentation dataset to study the properties of the proposed algorithm. While MNIST was originally constructed to facilitate research in image classification, in the form of recognising handwritten digits [23], it has found its use in segmentation task, which segment the digital from the background. It can be seen as a an image classification task, except that instead of classifying the whole image, we are classifying each pixel individually. MNIST dataset consists of 60,000 training and 10,000 testing examples, all of which are 28 $\times$ 28 grayscale images of digits from 0 to 9, and we derive the segmentation labels by thresholding the intensity values at 0.5.

ISBI2015 MS Lesion Segmentation Dataset: The ISBI2015 MS lesion challenge [7] was composed of 5 training and 14 testing subjects with 4 or 5 different image time-points per subject. All of the data were acquired on a 3.0 Tesla MRI scanner (Philips Medical Systems, Best, The Netherlands) with T1-w MPRAGE, FLAIR, T2-w sequences. On the challenge competition, each subject image was evaluated independently, which led to a final training and testing sets composed of 21 and 61 images, respectively. Manual delineations of MS lesions performed by two experts were included for each of the 21 training images. In our work, we extract the 2D slices from the 21 training images and use the slices from 3 subjects for training and the slices from another 2 subjects for testing, so the training data and the

testing data are not overlapped on same patient. We also applied data augmentation to increase the size of training and testing data. We perform data augmentation on-the-fly at batch time by multiplying the number of training samples by four following the next transformations [29]: for each mini-batch, all patches are first rotated with 180 degrees in the axial plane. From the original and rotated versions of the patches, new versions are computed by flipping those horizontally. Other rotations than 180 degrees are avoided, in order to roughly maintain the symmetry of the brain and avoid artificial rotations of brain structures.

Brain Tumour Segmentation Challenge 2019 (BraTS) Dataset: We also evaluate our model on a multi-class segmentation task on BraTS [24]. All BraTS scans are available as native (T1), post-contrast T1-weighted (T1Gd), T2-weighted (T2), T2-FLAIR volumes, and were acquired with different clinical protocols and various scanners from multiple institutions. All the imaging datasets have been segmented manually, by one to four raters, following the same annotation protocol, and their annotations were approved by experienced neuro-radiologists. We use all of the 259 high grade glioma (HGG) cases in training data. We extract each slice as 2D images and split them case-wise to have 1600 images for training, 300 for validation and 500 for testing. Pre-processing includes: concatenation of all of available modalities; centre cropping to 192×192; normalisation for each case and each modality.

Lung Image Database Consortium (LIDC-IDRI) Dataset: We also consider the LIDC-IDRI dataset which contains multiple annotations per input acquired from different clinical experts as the evaluation in practice. The dataset contains 1018 lung CT scans from 1010 lung patients with manual

lesion segmentations from four experts. For each scan, 4 radiologists provided annotation masks for lesions that they independently detected and considered to be abnormal. For our experiments, we use the same method in [22] to pre-process all scans. We split the dataset case-wise into a training (722 patients), validation (144 patients) and testing (144 patients). We then resampled the CT scans to $1mm \times 1mm$ in-plane resolution. We also centre cropped 2D images (180×180 pixels) around lesion positions, in order to focus on the annotated lesions. The lesion positions are those where at least one of the experts segmented a lesion. We assign 5000 images to the training, 1000 images to the validation and 1000 images to the testing. Since the dataset does not provide a single curated expert consensus label for each image, we created a "gold standard" by aggregating the labels via spatial STAPLE [5], a recent variant of the STAPLE framework employed in the creation of public medical image segmentation datasets e.g., ISLES [32], MSSeg [11], Gleason'19 [13] datasets. We further note that, as before, we assume labels are only available to the model during training, but not at test time, thus label aggregation methods cannot be applied on the test examples.

QSMSC at UCL Lesion Segmentation Dataset: Finally, we created a real-world dataset using 10 MS scans obtained from UCLH with manual segmentations from 4 different annotators (3 radiologists with different level skills and 1 expert to generate the expert consensus label). Although we have evaluated our model on both synthetic and public datasets, we would like to put our model into more realistic situation for evaluation. In this experiment, we create a MS lesion segmentation dataset using MS patients treated

Dataset	Learning Rate	Epoch	Batch Size	Augmentation	weight for regularisation (λ)
MNIST	1e-4	60	2	Random flip	0.7
MS	1e-4	55	2	Random flip	0.7
BraTS	1e-4	60	8	Random flip	1.5
LIDC-IDRI	1e-4	75	4	Random flip	0.9

Table 9: Hyper-parameters used for respective datasets.

with natalizumab at the National Hospital for Neurology and Neurosurgery, University College London Hospitals NHS Foundation Trust, London, UK, according to local, standard-of-care protocols, for whom at least one full brain scan pre- or post-initiation of natalizumab was available. The study had ethical and institutional approval at University College London Hospitals NHS Foundation Trust for consentless analysis of fully-anonymised, routinely collected data and was performed in accordance with the relevant guidelines and regulations. Our analysis used T1-weighted and fluid-attenuated inversion recovery (FLAIR) scans from individual patient with image size 150×256, having an average voxel resolution of $1 \times 1 \times 6mm^3$, which is typical of routine, standard-of-care, brain MR imaging in the UK. The 10 3D scans come from individual patient and we extract the 2D slices for our experiments. Finally, we have 1536 slices for training and 1024 slices for testing, where has no patient overlap on both sets.

7. Annotation Simulation Details

We generate synthetic noisy annotations from an assumed expert consensus label on MNIST, MS lesion and BraTS datasets, to demonstrate efficacy of the approach in an idealised situation where the expert consensus label is known. We simulate a group of 5 annotators of disparate

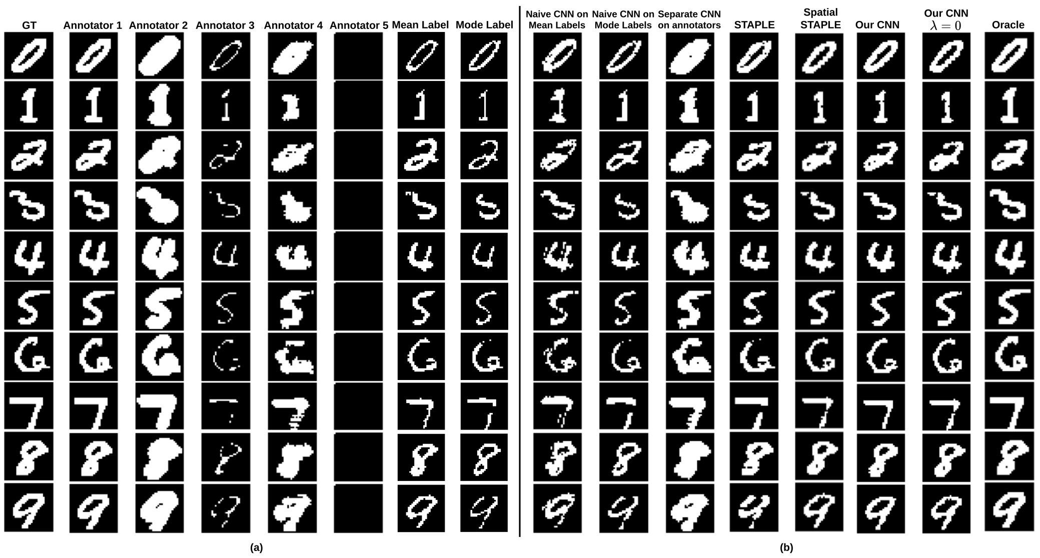

Figure 11: Visualisation of segmentation labels on MNIST dataset for single label per image: (a) expert consensus label and simulated annotator's segmentations (Annotator 1 - 5) and the corresponding mean label and mode label; (b) the predictions from the supervised models.

characteristics by performing morphological transformations (e.g., thinning, thickening, fractures, etc) on the expert consensus label, using Morpho-MNIST software [9]. In particular, *annotator 1* provides faithful segmentation ("good-segmentation") with approximate expert consensus label, *annotator 2* tends to over-segment ("over-segmentation"), *annotator 3* tends to under-segment ("under-segmentation"), *annotator 4* is prone to be a poor segmentation, which is simulated by combining small fractures and over-segmentation ("wrong-segmentation") and *annotator 5* always annotates everything as the background ("blank-segmentation"). To create synthetic noisy labels in multi-class scenario, we use a similar simulation to create noisy labels on the BraTS dataset. We first choose a target class and then ap-

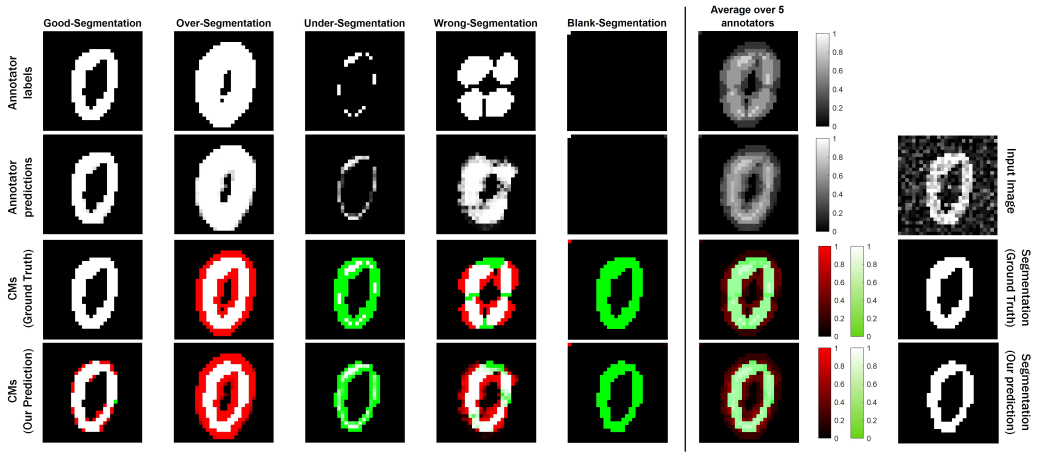

Figure 12: Visualisation of estimated expert consensus label and confusion matrices on MNIST datasets (Best viewed in colour: white is the true positive, green is the false negative, red is the false positive and black is the true negative. The background label is learned as true negative and false negative).

ply morphological operations on the provided expert consensus mask to create 4 synthetic noisy labels at different patterns, namely, over-segmentation, under-segmentation, wrong segmentation and good segmentation. We create training data by deriving labels from the simulated annotators. We also experimented with varying the levels of morphological operations on MNIST and MS lesion datasets, to test the robustness of our methods to varying degrees of annotation noise.

8. Implementation details

Our method is implemented in Pytorch [12]. All of the models were trained on a NVIDIA RTX 208 for at least 3 times with different random initialisations to compute the mean performance and its standard deviation (run 3 times of the experiments with the same initialization). The same

regularization strength is used across all the datasets. In practice, a small portion of data with clean labels (a fraction of test data) should be used to selected this hyper-parameter. The Adam [21] optimiser was used in all experiments with the default hyper-parameter settings. We also provide all of the hyper-parameters of the experiments for each data set in Table. 9. We also kept the training details the same between the baselines and our method.

Models	LIDC-IDRI DICE (%) (Dense Labels)	LIDC-IDRI DICE (%) (Single Label)
Mean labels	52.39	n/a
Mode labels	54.29	n/a
Naive CNN on mean labels	56.72 ± 0.61	48.36 ± 0.79
Naive CNN on mode labels	58.64 ± 0.47	48.36 ± 0.79
Probabilistic U-net [22]	61.26 ± 0.69	n/a
STAPLE [30]	69.34 ± 0.58	57.32 ± 0.87
Spatial STAPLE [5]	n/a	n/a
Ours with Global CMs	70.94 ± 0.19	63.25 ± 0.66
Ours without Trace	71.25 ± 0.12	66.95 ± 0.51
Ours	**74.12 ± 0.19**	**68.12 ± 0.48**
Oracle (Ours but with known CMs)	79.41 ± 0.17	n/a

Table 10: Comparison of segmentation accuracy and error of CM estimation for different methods trained with **dense labels** and **single label** (mean ± standard deviation), respectively. The best results are shown in bold. Numbers in bold indicate the best method that statistically ($p < 0.01$) better than other methods by computing the p values of paired t-tests on DICE metric. Note that we count out the Oracle from the model ranking as it forms a theoretical upper-bound on the performance where expert consensus label is known on the training data.

9. Proof of Theorem 1

We first show a specific case of Theorem 1 when there is only a single annotator, and subsequently extend it to the scenario with multiple annotators. Without loss of generality, we show the result for an arbitrary choice of a pixel in a given input image $\mathbf{x} \in \mathbb{R}^{W\times H\times C}$. Specifically, let us denote the estimated confusion matrix (CM) of the annotator at the $(i,j)^{\text{th}}$ pixel by $\hat{\mathbf{A}} := [\hat{\mathbf{A}}_{\phi}(\mathbf{x})_{ij}] \in [0,1]^{L\times L}$, and suppose the true class of this pixel is $k \in [1,...,L]$ i.e., $\mathbf{p}(\mathbf{x}) = \mathbf{e}_k$ where $\mathbf{e}_k$ denotes the kth elementary basis. Let $\hat{\mathbf{p}}_{\theta}(\mathbf{x})$ denote the L-dimensional estimated label distribution at the corresponding pixel (instead of over all the whole image).

Lemma 1. *If the annotator's segmentation probability is fully captured by the model for the $(i,j)^{th}$ pixel in image $\mathbf{x}$ i.e., $\hat{\boldsymbol{A}} \cdot \hat{\boldsymbol{p}}_{\theta}(\boldsymbol{x}) = \boldsymbol{A} \cdot \boldsymbol{p}(\boldsymbol{x})$, and both $\hat{\boldsymbol{A}}$, $\boldsymbol{A}$ satisfy that $a_{kk} > a_{kj}$ for $j \neq k$ and $\hat{a}_{ii} > \hat{a}_{ij}$ for all i,j such that $j \neq i$, then $tr(\hat{\boldsymbol{A}})$ is minimised when $\hat{\boldsymbol{A}} = \boldsymbol{A}$. Furthermore, if $tr(\hat{\boldsymbol{A}}) = tr(\boldsymbol{A})$, then the true label is fully recovererd i.e., $\hat{\boldsymbol{p}}_{\theta}(\boldsymbol{x}) = \boldsymbol{p}(\boldsymbol{x})$ and the kth column in $\hat{\boldsymbol{A}}$, $\boldsymbol{A}$ are the same.*

Proof 9.1. *We first show that the kth diagonal element in $\boldsymbol{A}$ is smaller than or equal to its estimate in $\hat{\boldsymbol{A}}$. Since $\boldsymbol{p}(\boldsymbol{x}) = \mathbf{e}_k$ is a one-hot vector, $\hat{\boldsymbol{A}} \cdot \hat{\boldsymbol{p}}_{\theta}(\boldsymbol{x}) = \boldsymbol{A} \cdot \boldsymbol{p}(\boldsymbol{x})$ holds and $\hat{a}_{kk} > \hat{a_{kj}} \forall j \neq k$, it follows that:*

$$a_{kk} = \Big\langle [\hat{a}_{k1}, ..., \hat{a}_{kL}], \quad \hat{\boldsymbol{p}}_{\theta}(\boldsymbol{x}) \Big\rangle \tag{7}$$

$$\leq \Big\langle [\hat{a}_{kk}, ..., \hat{a}_{kk}], \quad \hat{\boldsymbol{p}}_{\theta}(\boldsymbol{x}) \Big\rangle = \hat{a}_{kk}. \tag{8}$$

The possibility of equality in the above comes from the fact that all entries in $\hat{\boldsymbol{p}}_\theta(\boldsymbol{x})$ except the kth element could be zeros. Now, the assumption that there is a single ground truth label k for the $(i,j)^{th}$ pixel means that all the values of the true CM, $\boldsymbol{A}$ are uniformly equal to $1/L$ except the kth column. In addition, since the diagonal dominance of the estimated CM means each $\hat{a}_{ii}$ is at least $1/L$, we have that

$$tr(\boldsymbol{A}) = \frac{L-1}{L} + a_{kk} \leq \sum_{j \neq k} \hat{a}_{jk} + \hat{a}_{kk} = tr(\hat{\boldsymbol{A}}).$$

It therefore follows that when $\hat{\boldsymbol{A}} = \boldsymbol{A}$ holds, the trace of $tr(\hat{\boldsymbol{A}})$ is the smallest. Now, we show that when this holds i.e., $tr(\boldsymbol{A}) = tr(\hat{\boldsymbol{A}})$, then the kth columns of the two matrices match up.

By way of contradiction, let us assume that there exists a class $k' \neq k$ for which the estimated label probability is non-zero i.e., $\hat{p}_{k'} := [\hat{\boldsymbol{p}}_\theta(\boldsymbol{x})]_{k'} > 0$. This implies that $1 - \hat{p}_k > 0$. From eq. (8), if the trace of $\boldsymbol{A}$ and $\hat{\boldsymbol{A}}$ are the same, then $a_{kk} = \hat{a}_{kk}$ also holds and thus we have $\hat{a}_{kk} = \sum_j \hat{a}_{kj}\hat{p}_j$. By rearranging this equality and dividing both sides by $1 - \hat{p}_k$, we obtain $\hat{a}_{kk} = \sum_{j \neq k} \frac{\hat{p}_j}{1-\hat{p}_k}\hat{a}_{kj}$. Now, as we have $\hat{a}_{kk} > \hat{a}_{kj}, j \neq k$, it follows that

$$\hat{a}_{kk} < \hat{a}_{kk} \sum_{j \neq k} \frac{\hat{p}_j}{1 - \hat{p}_k} = \hat{a}_{kk}$$

which is false. Therefore, the trace quality implies $\hat{p}_k = 1$ and thus from $\hat{\boldsymbol{A}} \cdot \hat{\boldsymbol{p}}_\theta(\boldsymbol{x}) = \boldsymbol{A} \cdot \boldsymbol{p}(\boldsymbol{x})$, we conclude that the kth columns of $\hat{\boldsymbol{A}}$ and $\boldsymbol{A}$ are the same.

We note that the equivalent result for the expectation of the annotator's CM over the data population was provided in [27] and [28]. The main differ-

ence is, as described in the main text, that we show a slightly weaker version of their result in a sample-specific scenario.

Now, we show that the main theorem follows naturally from the above lemma. As a reminder, we recite the theorem below.

Theorem 1. *For the* $(i,j)^{th}$ *pixel in a given image* $\mathbf{x}$*, we define the mean confusion matrix (CM)* $\boldsymbol{A}^* := \sum_{r=1}^{R} \pi_r \hat{\boldsymbol{A}}^{(r)}$ *and its estimate* $\hat{\boldsymbol{A}}^* := \sum_{r=1}^{R} \pi_r \hat{\boldsymbol{A}}^{(r)}$ *where* $\pi_r \in [0,1]$ *is the probability that the annotator* r *labels image* $\mathbf{x}$*. If the annotator's segmentation probabilities are perfectly modelled by the model for the given image i.e.,* $\hat{\boldsymbol{A}}^{(r)} \hat{\boldsymbol{p}}_\theta(\boldsymbol{x}) = \boldsymbol{A}^{(r)} \boldsymbol{p}(\boldsymbol{x}) \forall r = 1, ..., R$*, and the average true confusion matrix* $\boldsymbol{A}^*$ *at a given pixel and its estimate* $\hat{\boldsymbol{A}}^*$ *satisfy that* $a^*_{kk} > a^*_{kj}$ *for* $j \neq k$ *and* $\hat{a}^*_{ii} > \hat{a}^*_{ij}$ *for all* i, j *such that* $j \neq i$*, then* $\boldsymbol{A}^{(1)}, ..., \boldsymbol{A}^{(R)} = \text{argmin}_{\hat{\boldsymbol{A}}^{(1)}, ..., \hat{\boldsymbol{A}}^{(R)}} \left[tr(\hat{\boldsymbol{A}}^*) \right]$ *and such solutions are* ***unique*** *in the* k^{th} *columns where* k *is the correct pixel class.*

Proof 9.2. *A direct application of Lemma 1 shows firstly that* $tr(\hat{\boldsymbol{A}}^*)$ *is minimised when* $\hat{\boldsymbol{A}}^{(r)} = \boldsymbol{A}^{(r)}$ *for all* $r = 1, ..., R$ *(since that ensures* $\boldsymbol{A}^* = \hat{\boldsymbol{A}}^*$*). Secondly, it implies that minimising* $tr(\hat{\boldsymbol{A}}^*)$ *yields* $\hat{\boldsymbol{p}}_\theta(\boldsymbol{x}) = \boldsymbol{p}(\boldsymbol{x})$*. Because we assume that annotators' noisy labels are correctly modelled i.e.,* $\hat{\boldsymbol{A}}^{(r)} \hat{\boldsymbol{p}}_\theta(\boldsymbol{x}) = \boldsymbol{A}^{(r)} \boldsymbol{p}(\boldsymbol{x}) \forall r = 1, ..., R$*, it therefore follows that the* k^{th} *column in* $\hat{\boldsymbol{A}}^{(r)}$ *and* $\boldsymbol{A}^{(r)}$ *are the same.*

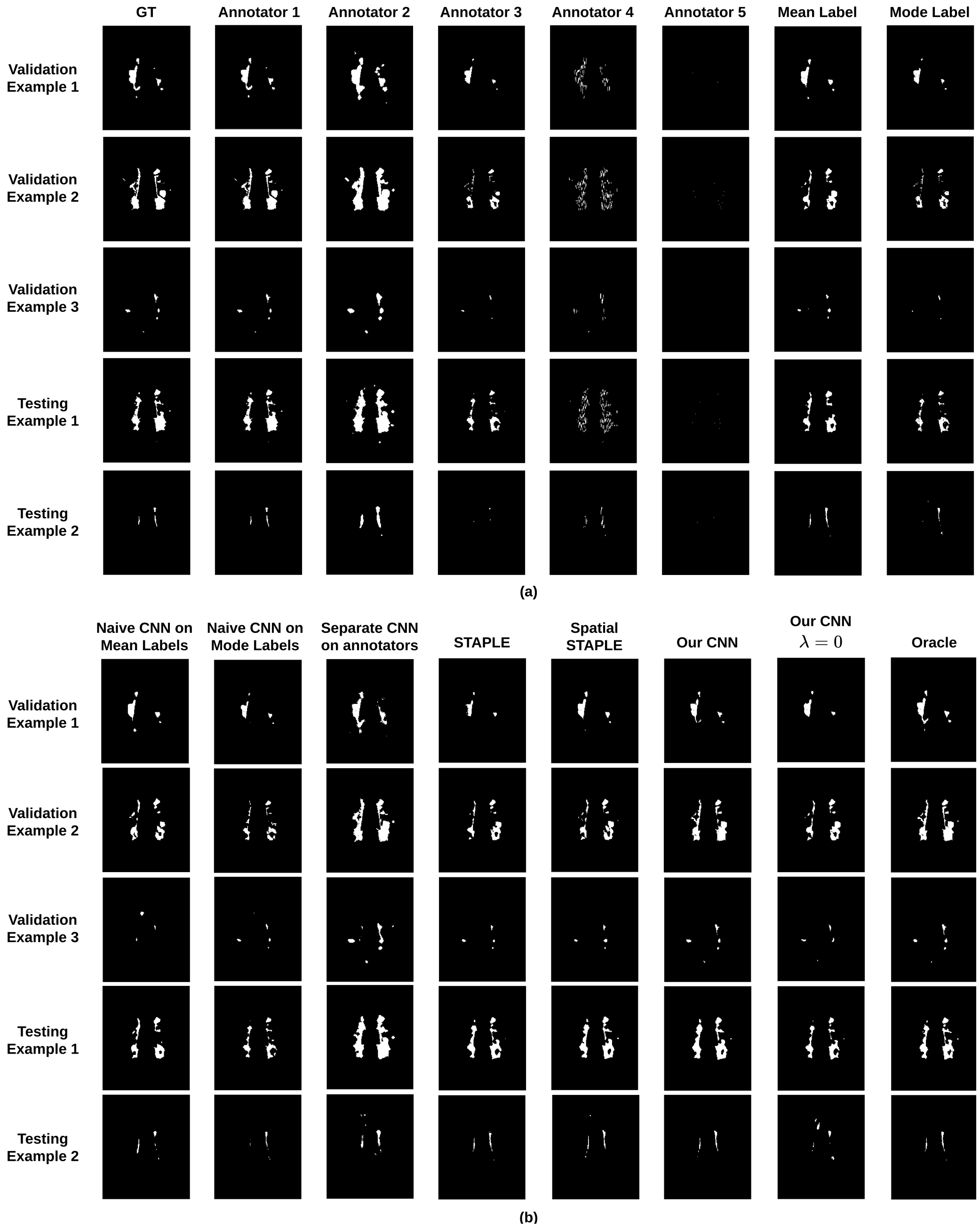

Figure 13: Visualisation of segmentation labels on ISBI 2015 challenge MS segmentation dataset for single label per image: (a) expert consensus label and simulated annotator's segmentations (Annotator 1 - 5) and the corresponding mean label and mode label; (b) the predictions from the supervised models

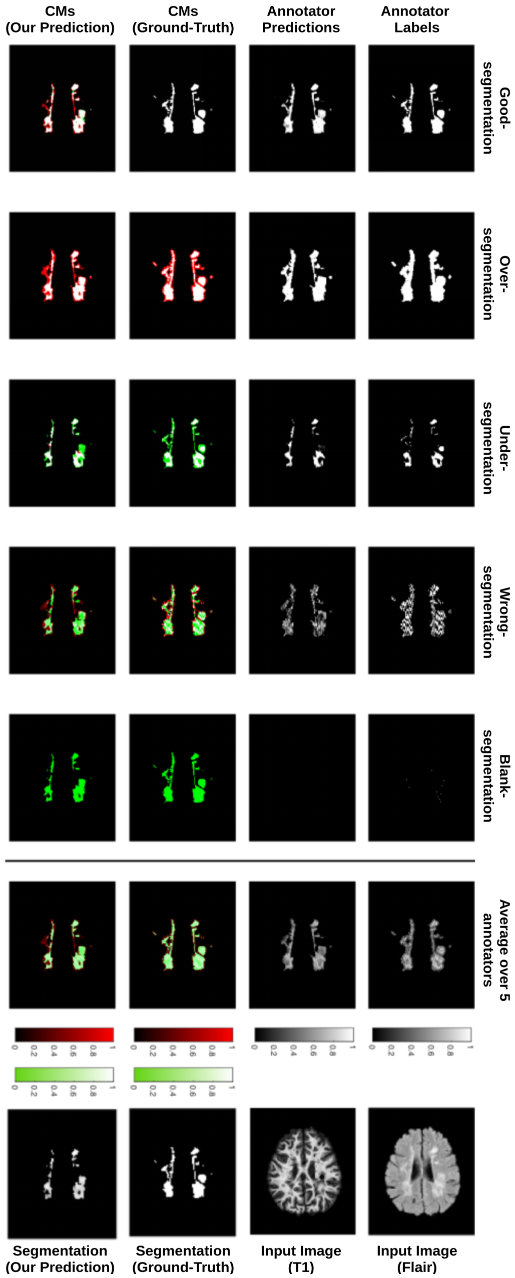

Figure 14: Visualisation of estimated expert consensus label and confusion matrices on ISBI 2015 challenge MS segmentation datasets (Best viewed in colour: white is the true positive, green is the false negative, red is the false positive and black is the true negative. The background label is learned as true negative and false negative.)

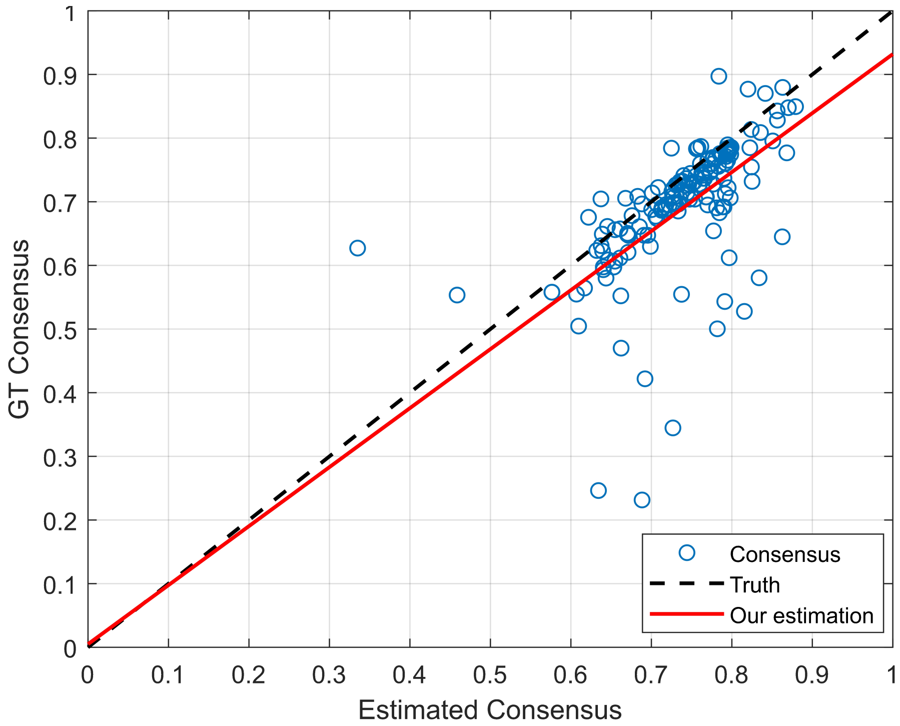

Figure 15: The consensus level amongst the estimated annotators is plotted against the expert consensus label on LIDC-IDRI dataset. The strong positive linear correlation shows that the variation in the inter-reader variability on different input examples (e.g., some examples are more ambiguous than others) is captured well. We do note, however, that the inter-reader variation seems more under-estimated for "easy" (i.e., higher consensus) samples.

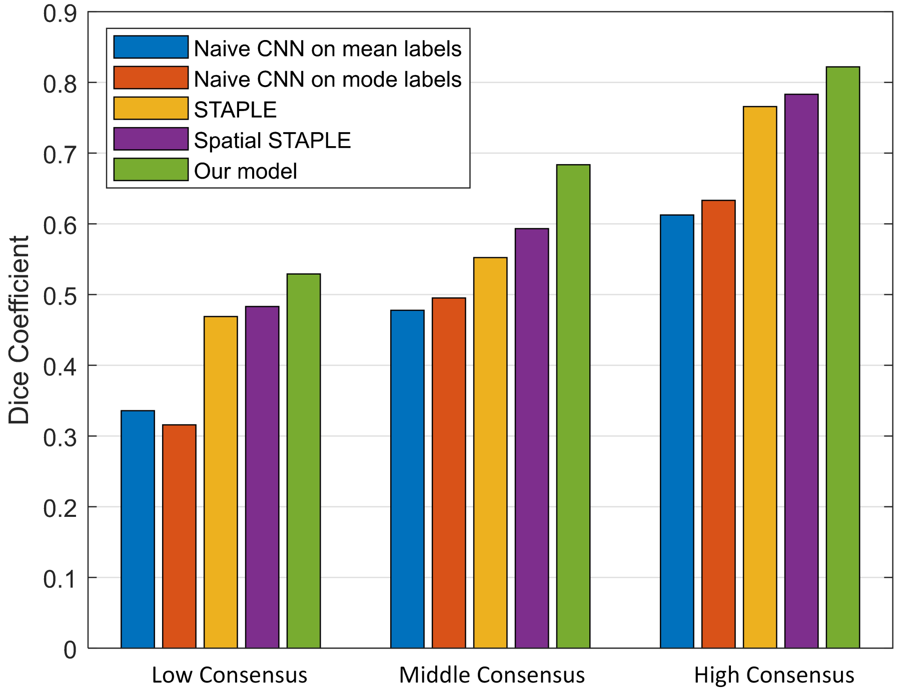

Figure 16: Segmentation performance on 3 different subgroups of the LIDC-IDRI dataset with varying levels of inter-reader agreement. Our method shows *consistent* improvement over the baselines and the competing methods in all groups, showing its enhanced ability to segment challenging examples (i.e., low-consensus cases).
